# A natural polymorphism of *Mycobacterium tuberculosis* in the *esxH* gene disrupts immunodomination by the TB10.4-specific CD8 T cell response

**DOI:** 10.1371/journal.ppat.1009000

**Published:** 2020-10-19

**Authors:** Rujapak Sutiwisesak, Nathan D. Hicks, Shayla Boyce, Kenan C. Murphy, Kadamba Papavinasasundaram, Stephen M. Carpenter, Julie Boucau, Neelambari Joshi, Sylvie Le Gall, Sarah M. Fortune, Christopher M. Sassetti, Samuel M. Behar

**Affiliations:** 1 Immunology and Microbiology Program, Graduate School of Biomedical Science, University of Massachusetts Medical School, Worcester, Massachusetts, United States of America; 2 Department of Microbiology and Physiological Systems, University of Massachusetts Medical School, Worcester, Massachusetts, United States of America; 3 Department of Immunology and Infectious Diseases, Harvard T.H. Chan School of Public Health, Boston, Massachusetts, United States of America; 4 Ragon Institute of Massachusetts General Hospital, Massachusetts Institute of Technology and Harvard University, Cambridge, MA, United States of America; Portland VA Medical Center, Oregon Health and Science University, UNITED STATES

## Abstract

CD8 T cells provide limited protection against *Mycobacterium tuberculosis* (Mtb) infection in the mouse model. As Mtb causes chronic infection in mice and humans, we hypothesize that Mtb impairs T cell responses as an immune evasion strategy. TB10.4 is an immunodominant antigen in people, nonhuman primates, and mice, which is encoded by the *esxH* gene. In C57BL/6 mice, 30–50% of pulmonary CD8 T cells recognize the TB10.4_4−11_ epitope. However, TB10.4-specific CD8 T cells fail to recognize Mtb-infected macrophages. We speculate that Mtb elicits immunodominant CD8 T cell responses to antigens that are inefficiently presented by infected cells, thereby focusing CD8 T cells on nonprotective antigens. Here, we leverage naturally occurring polymorphisms in *esxH*, which frequently occur in lineage 1 strains, to test this “decoy hypothesis”. Using the clinical isolate 667, which contains an EsxH^A10T^ polymorphism, we observe a drastic change in the hierarchy of CD8 T cells. Using isogenic Erd.EsxH^A10T^ and Erd.EsxH^WT^ strains, we prove that this polymorphism alters the hierarchy of immunodominant CD8 T cell responses. Our data are best explained by immunodomination, a mechanism by which competition for APC leads to dominant responses suppressing subdominant responses. These results were surprising as the variant epitope can bind to H2-K^b^ and is recognized by TB10.4-specific CD8 T cells. The dramatic change in TB10.4-specific CD8 responses resulted from increased proteolytic degradation of A10T variant, which destroyed the TB10.4_4-11_epitope. Importantly, this polymorphism affected T cell priming and recognition of infected cells. These data support a model in which nonprotective CD8 T cells become immunodominant and suppress subdominant responses. Thus, polymorphisms between clinical Mtb strains, and BCG or H37Rv sequence-based vaccines could lead to a mismatch between T cells that are primed by vaccines and the epitopes presented by infected cells. Reprograming host immune responses should be considered in the future design of vaccines.

## Author summary

An important question for vaccine developers is the relative potency of CD4 vs. CD8 T cells against Mtb, as strategies differ for eliciting these different T cell subsets. Despite robust antigen-specific pulmonary CD8 T cell responses, CD4 T cells mediate more protection than CD8 T cells in the murine model. Most CD8 T cells recognize a single antigen, TB10.4, which is encoded by the *esxH* gene. Based on finding that TB10.4_4−11_-specific CD8 T cells poorly recognize Mtb-infected macrophages, we hypothesized that Mtb evades detection by CD8 T cells and focuses the CD8 T cell response on non-protective antigen. We termed these antigens “decoy antigens.” To test this hypothesis, we took advantage of a natural variant of the *esxH* gene, which contains an A10T polymorphism within the TB10.4_4−11_ epitope. This polymorphism drastically alters the hierarchy of CD8 T cell responses elicited by Mtb. These data suggest that immunodomination by the TB10.4 epitope acts to suppress subdominant CD8 T cell responses to other Mtb antigens, impairing the CD8 T cell response to other Mtb antigens, some of which might be presented by Mtb-infected macrophages and be targets of protective immunity. Importantly, this single amino acid polymorphism, which does not significantly alter MHC-binding or T cell recognition, alters the half-life of the epitope and consequently, has a profound effect on CD8 T cell priming and recognition of infected cells. These data also provide a mechanism that could be exploited to manipulate the hierarchy of immunodominant responses.

## Introduction

Tuberculosis (TB), a disease caused by *Mycobacterium tuberculosis* (Mtb), is the leading cause of death from an infectious disease [1]. Mtb infects myeloid cells, largely evades humoral immunity and persists intracellularly by inhibiting vesicular trafficking and phagolysosome fusion [2]. The protective role of CD4 T cells is a reflection of bacillary occupation of the phagosome as Mtb protein antigens in the vacuolar compartment are processed and sampled by class II MHC. While CD8 T cells predominantly recognize peptides generated in the cytosol and sampled by class I MHC, vacuolar antigens can enter the class I MHC pathway through a process called cross-presentation [3]. Robust CD8 T cell responses are generated when uninfected DC cross-present Mtb antigens that are acquired by the uptake of apoptotic vesicles or exosomes derived from infected cells [4]. However, the role of CD8 T cells in host protection is controversial. On the one hand, CD8 T cells confer far less protection than CD4 T cells in the murine aerosol TB model [5]. Yet, recent data from the NHP TB model indicates that CD8 T cell responses correlate with protection elicited by vaccination [6–8]. The ability to improve vaccine efficacy by targeting CD8 T cells would be an important advance. In the mouse model, CD8 T cells make only a modest contribution to protective immunity despite a robust immune response (see below). To understand why protection mediated by CD8 T cells is suboptimal in the mouse model, we have advanced the hypothesis that some immunodominant Mtb antigens act as decoys by eliciting CD8 T cell responses that inefficiently recognize infected cells [9]. Understanding the mechanisms that interfere with CD8-mediated protection could provide new strategies for the development of protective vaccines.

*EsxH* is an essential gene that is part of the ESX3 type VII secretion system [10]. The *esxH* gene encodes the protein TB10.4, which elicits both CD4 and CD8 T cell responses in human and mice, and has been incorporated into vaccines undergoing clinical trials (e.g., AERAS-402) [11]. After Mtb aerosol infection of C57BL/6J mice, 30–50% of pulmonary CD8 T cells are specific to the TB10.4_4−11_ epitope [12]. TB10.4_4−11_-specific CD8 T cells mediate protection when transferred to immunocompromised mice, but TB10.4_4−11_-immunization did not protect immunocompetent mice against Mtb challenge [13, 14]. Recently, we found that TB10.4_4−11_-specific CD8 T cells do not recognize Mtb-infected macrophages, which is difficult to reconcile with the immunodominance of TB10.4-specific CD8 T cell response [9].

Why the CD8 T cell response to TB10.4 is immunodominant is unclear [13]. Immunodominant CD8 T cell responses are frequently seen after viral infection [15]. One mechanism observed during HIV, CMV and poxvirus infection, among others, is immunodomination [16–19]. Immunodomination is defined as a dominant CD8 T cell response which positively reinforces itself by inhibiting CD8 T cell responses to other sub-dominant epitopes [20, 21]. The inability of immunodominant TB10.4-specific CD8 T cell response to recognize Mtb-infected macrophages suggests that this type of response could be an evasion strategy that benefits Mtb. By stimulating a dominant CD8 T cell response that cannot recognize infected macrophages, TB10.4 might act as a decoy antigen, and prevent the generation of other Mtb-specific CD8 T cell responses that could potentially mediate protection, while at the same time, creating an inflammatory environment that promotes bacterial transmission.

To investigate these questions, we examined the highly polymorphic nature of *esxH*, and identified a “hotspot” in the TB10.4_4−11_ epitope, particularly in lineage 1. We leveraged naturally occurring polymorphisms among clinical isolates to study immunodomination and immune evasion. By using the clinical isolate 667, which has a polymorphism at the tenth amino acid in the TB10.4 protein (i.e., A10T), we show that the hierarchy of the Mtb-specific CD8 T cell response is driven by immunodomination. We then considered whether this change in hierarchy could result from altered presentation of the TB10.4_4−11_ epitope or arise from a change in function of the TB10.4 protein. We prove that the alteration in immunodominance was due to the variation in *esxH* by developing a set of isogenic Erdman strains with the same A10T polymorphism. Finally, we discovered that the A10T polymorphism changes the processing of the TB10.4_4−11_ epitope and leads to its accelerated destruction. This change in the epitope sequence, which does not significantly alter MHC-binding or T cell recognition, alters the half-life of the epitope and consequently, has profound effects on CD8 T cell priming and immunodomination.

## Results

### The TB10.4_4−11_ epitope is a hotspot for polymorphisms in the *esxH* gene of Mtb

**Fig 1:**
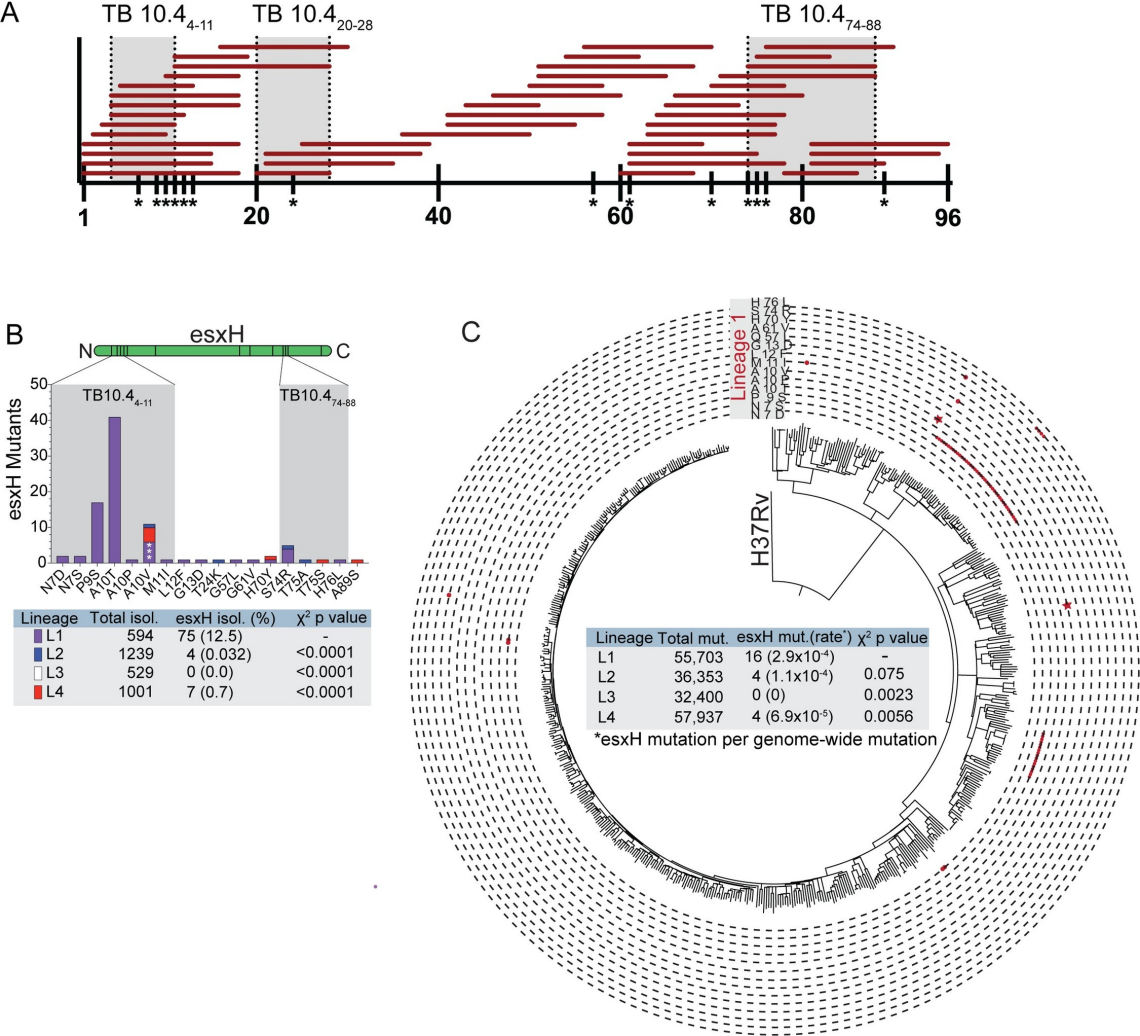
The *esxH* gene is highly polymorphic among clinical Mtb isolates. (A) Reported TB10.4 epitopes recognized by human T cells from the IEDB database (www.iedb.org). Y axis represents the length of the 96 amino-acid TB10.4 protein. Each horizontal red bar spans an individual reported epitope. Grey shaded areas represent mouse TB10.4-specific T cell epitopes: H2-K^b^-restricted TB10.4_4−11_, H2-K^d^-restricted TB10.4_20−28_, and I-A^d^-restricted TB10.4_74−88_. (B) The locations of EsxH polymorphisms are represented along the linear sequence of the protein (green), with the number of isolates containing each polymorphism based on the whole genome sequences from Holt et al and Walker et al [25, 26]. Regions of the protein with a high frequency of polymorphisms are shaded grey. The bar color corresponds to the different Mtb lineages: lineage 1 (L1), purple; L2, blue; L3, white; and, L4, red. The table shows the total numbers of polymorphisms in each lineage, and the χ^2^ and p values compared to L1. (C) Whole-genome SNP-based phylogenies represent the polymorphisms across all clinical isolates within lineage 1. Each circumference line signifies a distinct polymorphism indicated in the grey box labeled “Lineage 1.” Each red dot cluster represents each time the indicated polymorphism evolved independently. Red stars designate the A10V polymorphism which evolve separately three times. The table shows the acquisition rate of esxH variations based on the identification of genome-wide SNP events within each phylogeny and the number of events which were within esxH.

Numerous human T cell epitopes in the TB10.4 protein, which is encoded by *esxH* gene, have been identified from individuals with diverse HLA haplotypes (Fig 1A). In addition, three immunodominant epitopes have been identified in C57BL/6 and BALB/c mice (shaded grey, Fig 1A) [12, 22, 23]. As *esxH* is an essential gene required for iron homeostasis and virulence, and its deletion leads to attenuation *in vivo* [24], it was not feasible to test the “decoy hypothesis” by *esxH* deletion. Therefore, we investigated whether naturally occurring polymorphisms in virulent Mtb strains could be leveraged to study the how immunodominance affects host immunity.

The frequency of *esxH* polymorphisms among 3,363 *M*. *tuberculosis* clinical isolates from Holt *et al* and Walker *et al*, spanning lineages 1–4 (i.e., L1, L2, L3 and L4), was determined by whole-genome sequencing [25, 26]. A total of 86 isolates contained non-synonymous polymorphisms in *esxH* (2.56%), of which the majority (77 isolates) resulted in amino acids changes between positions N7 and G13 (Fig 1B, S1 Table). Clinical isolates with *esxH* polymorphisms were not randomly distributed among the four *M*. *tuberculosis* lineages. In particular, 75 of 594 (12.5%) isolates belonging to L1 contained *esxH* polymorphism, while the frequency in lineages L2, L3 and L4 were significantly lower with 4 of 1239 (0.32%), 0 of 529 (0%), and 7 of 1001 (0.7%), respectively (two-sided chi-square: L1 vs L2, χ^2^ = 147.4, p < 0.0001; L1 vs L3, χ^2^ = 71.57, p < 0.0001; L1 vs L4, χ^2^ = 108.7, p < 0.0001).

To understand the evolution of *esxH* variants, we constructed whole-genome SNP-based phylogenies for each lineage separately (Fig 1C). By mapping the genome-wide SNP alignment back to the phylogenetic tree, we could identify 55,703 SNP mutation events within the L1 phylogeny, of which 16 were within *esxH*. Assuming that each lineage was equally likely to alter *esxH* by chance, the number of variations in L1 was significantly higher than expected when compared with L3 (0 of 32400 mutations, two-sided chi-square, χ^2^ = 9.308, p = 0.0023) and L4 (4 of 57937 mutations, two-sided chi-square, χ^2^ = 7.684, p = 0.0056). The trend was consistent compared with L2 (4 of 36353 mutations, χ^2^ = 1.783, p = 0.075). Overall, these data suggest that L1 isolates are more likely to acquire *esxH* polymorphisms compared with the modern L2, L3 and L4 strains. We further calculated the number of times each SNP within L1 evolved independently by mapping the *esxH* variations back on the phylogeny. Each *esxH* variant evolved once except for A10V which evolved 3 times and could be found in one clade of four isolates and two unrelated isolates (purple stars, Fig 1C). Among the *esxH* variants, A10T is the most abundance which were found in 41 isolates. Since all of these clinical strains were by definition virulent, we elected to study the mechanism and the impact of the A10T polymorphism on immunodomination and immune evasion.

### The clinical isolate 667 and Erdman elicit different hierarchies of antigen-specific CD8 T cells

To determine whether *esxH* polymorphisms affect T cell responses *in vivo*, we infected C57BL/6J mice with the clinical isolate 667 [27]. 667 was selected because it has a single nonsynonymous polymorphism in *esxH* that results in the amino acid substitution A10T, which is the most frequent variation among the clinical isolates. Although it has ~1,700 SNPs compared to Erdman or the laboratory reference strain H37Rv, there are no differences among the genes that encode the major antigens ESAT6, Ag85B, CFP10, and MTB32a, which we use to measure T cell responses in the mouse tuberculosis model.

**Fig 2:**
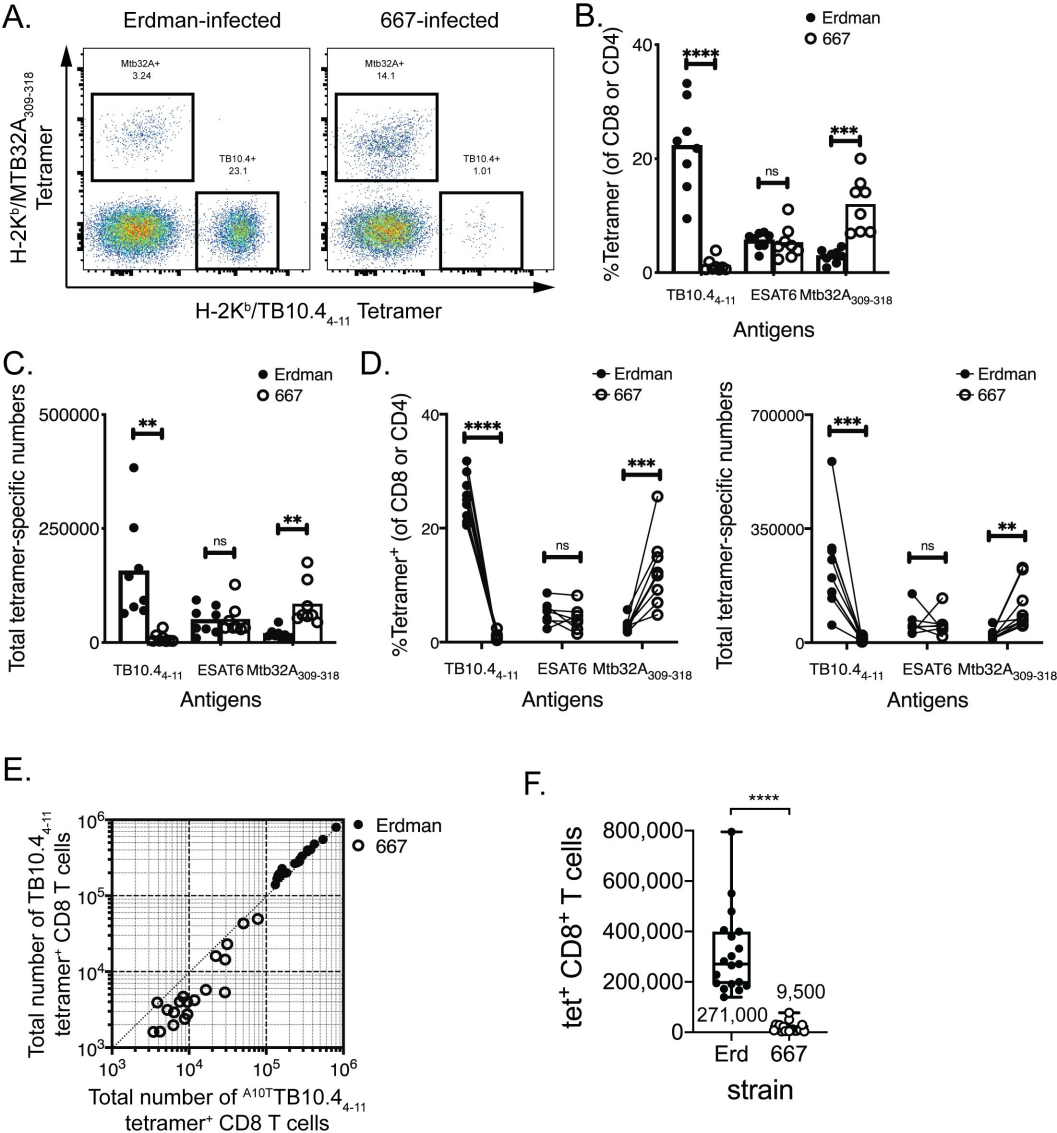
The hierarchy of immunodominant CD8 T cell responses after Erdman vs. 667 infection. Lung T cell responses were evaluated five weeks after infection of C57BL/6J mice with ~100 aerosolized Erdman or 667 bacilli. (A) Representative flow cytometry plots of MTB32a_309-318_ and TB10.4_4−11_ tetramer staining of pulmonary CD8 T cells from Erdman- or 667-infected C57BL/6J mice. The frequency (B) or absolute number (C) of TB10.4- or MTB32a-specific CD8, or ESAT6-specific CD4 T cells in the lungs of infected mice after Erdman or 667 infection. (D) The frequency (left) and total numbers (right) of Mtb-specific CD4 or CD8 T cells, from nine independent paired Erdman (closed symbols) and 667 (open symbols) infections. Each point is the average of 4–8 mice and lines connect the paired infections. (E) The number of TB10.4-specific CD8 T cells in the lungs of mice infected with Erdman (closed symbols) or 667 (open symbols) was quantified by staining the different cell populations using the TB10.4_4−11_/K^b^ tetramer (i.e., loaded with IMYNYPAM) or the ^A10T^TB10.4_4−11_/K^b^ tetramer (i.e., loaded with IMYNYPTM) separately using individual mice from 4 independent paired infections, 38 mice in total. The data was pooled and analyzed. The diagonal line is the line of unity for this analysis. (F) Absolute numbers of TB10.4-specific CD8 T cells in the lungs of mice infected with Erdman or 667. The number of cells was determined by staining with the TB10.4_4−11_/K^b^ tetramer (i.e., loaded with IMYNYPAM) or the ^A10T^TB10.4_4−11_/K^b^ tetramer (i.e., loaded with IMYNYPTM). Nine independent experiments were performed and analyzed 4–6 weeks post infection, with a total of 96 mice. Statistical testing by a two-tailed, unpaired Student’s T test. **, p<0.01; ***, p<0.005; and ****, p<0.0001.

Following aerosol infection with Erdman, the frequency of antigen-specific T cells was monitored by flow cytometry using tetramers of peptide-loaded MHC molecules. When the cells were stained with the TB10.4_4−11_/K^b^ tetramer corresponding to the IMYNYPAM epitope (hereafter referred to as “WT”), 26% of lung CD8 T cells were specific for TB10.4_4−11_; in contrast, fewer than 1% of lung CD8 T cells recognized TB10.4_4−11_ after 667 infection (Fig 2A and 2B). Not only was the frequency of TB10.4_4−11_-specific CD8 T cells diminished after 667 infection, but there was also a reduction in the absolute number of TB10.4_4−11_-specific CD8 T cells, a decrease that persisted during the course of infection (Fig 2C, S1A Fig). The frequency and number of ESAT6-specific CD4 T cells elicited by the two bacterial strains was similar (Fig 2B and 2C). Importantly, the frequency and number of MTB32a_309-318_-specific CD8 T cells was significantly greater after 667 infection, relative to Erdman infection (Fig 2A–2C, S1B Fig). These differences were observed in nine independent paired infections, which included 96 individually analyzed mice (Fig 2D). Antigen-specific CD8 T cells expressed high levels of KLRG1 and low levels of CD127, independently of the bacterial strain, which was consistent with an effector cell phenotype (S1C Fig).

We next considered whether the reduction in the TB10.4_4−11_-specific CD8 T cell response observed after 667 infection could be arise from a change in the fine specificity of TB10.4_4−11_-specific CD8 T cells in 667 infected mice such that they preferentially recognized the A10T epitope. To test this possibility, we compared ^WT^TB10.4_4−11_/K^b^ tetramers to tetramers loaded with the “A10T” variant peptide IMYNYPTM (hereafter referred to as “^A10T^TB10.4_4−11_”). Direct comparison of the two tetramers showed that both identified in similar number of TB10.4_4−11_-specific CD8 T cells in Erdman-infected mice (Fig 2E, filled symbols). In contrast, in 667-infected mice, the ^WT^TB10.4_4−11_/K^b^ tetramer underestimated the number of TB10.4_4−11_-specific CD8 T cells (Fig 2E, open symbols). This suggested that two populations of T cells elicited by Erdman and 667 differed in the avidity for the peptide/MHC complex. To determine the magnitude of this effect, we performed competitive tetramer staining. The ^WT^TB10.4_4−11_/K^b^ tetramers, but not the ^A10T^TB10.4_4−11_/K^b^ tetramers, bound CD8 T cells elicited by Erdman suggesting higher avidity interaction of the former (S2A Fig). In contrast, both tetramers bound to 667-elicited TB10-specific CD8 T cells, although there was a clear preference for the ^A10T^TB10.4_4−11_/K^b^ tetramer despite the ability of both tetramers to bind independently (S2B Fig). Hereafter we used the ^A10T^TB10.4_4−11_/K^b^ tetramer to quantify TB10.4_4−11_-specific CD8 T cells after 667 infection. The number of TB10.4_4−11_-specific CD8 T cells was 271,000 ± 163,000 after Erdman infection compared to 9,500 ± 19,000 after 667 infection (Fig 2F, p<0.0001, median ± SD). Thus, the CD8 T cell response to TB10.4_4−11_ after 667 infection was largely abolished.

Since we observed a loss of the dominant TB10.4_4−11_ response during 667 infection, we sought to determine whether a cryptic epitope of TB10.4 would emerge after 667 infection. Cryptic epitopes are ones that do not ordinarily elicit a T cell response but can induce T cells if immunodominance is altered as has been described for ESAT6 [28]. We screened a peptide library of the TB10.4 protein plus other known epitopes using T cells from 667-infected or Erdman-infected mice but did not identify any new epitopes (S2 Table, S3 Fig). We also sought to determine whether the EsxH^A10T^ polymorphism affected the K^d^-restricted CD8 T cell response to TB10.4_20−28_ or the I-A^d^-restricted CD4 T cell response to TB10.4_74−88_ in Mtb-infected BALB/c mice [23, 29]. The TB10.4_20−28_-specific CD8 T cell response was diminished in 667 vs. Erdman infected mice only in one of three experiments (S4 Fig). The TB10.4_74−88_-specific CD4 T cell response was of similar magnitude after 667 or Erdman infection in all experiments (S4 Fig). Thus, these data show that 667 infection elicits a hierarchy of Mtb-specific CD8 T cells that quantitatively differs from the reproducible hierarchy elicited by Erdman infection in C57BL/6 mice. These data are consistent with immunodomination being the mechanism by which immunodominant TB10.4_4−11_-specific CD8 T cell response suppresses other subdominant responses and indicates that the EsxH^A10T^ polymorphism can be used to perturb the hierarchy of Mtb antigens recognized by CD8 T cells.

### 667 is less fit than Erdman

**Fig 3:**
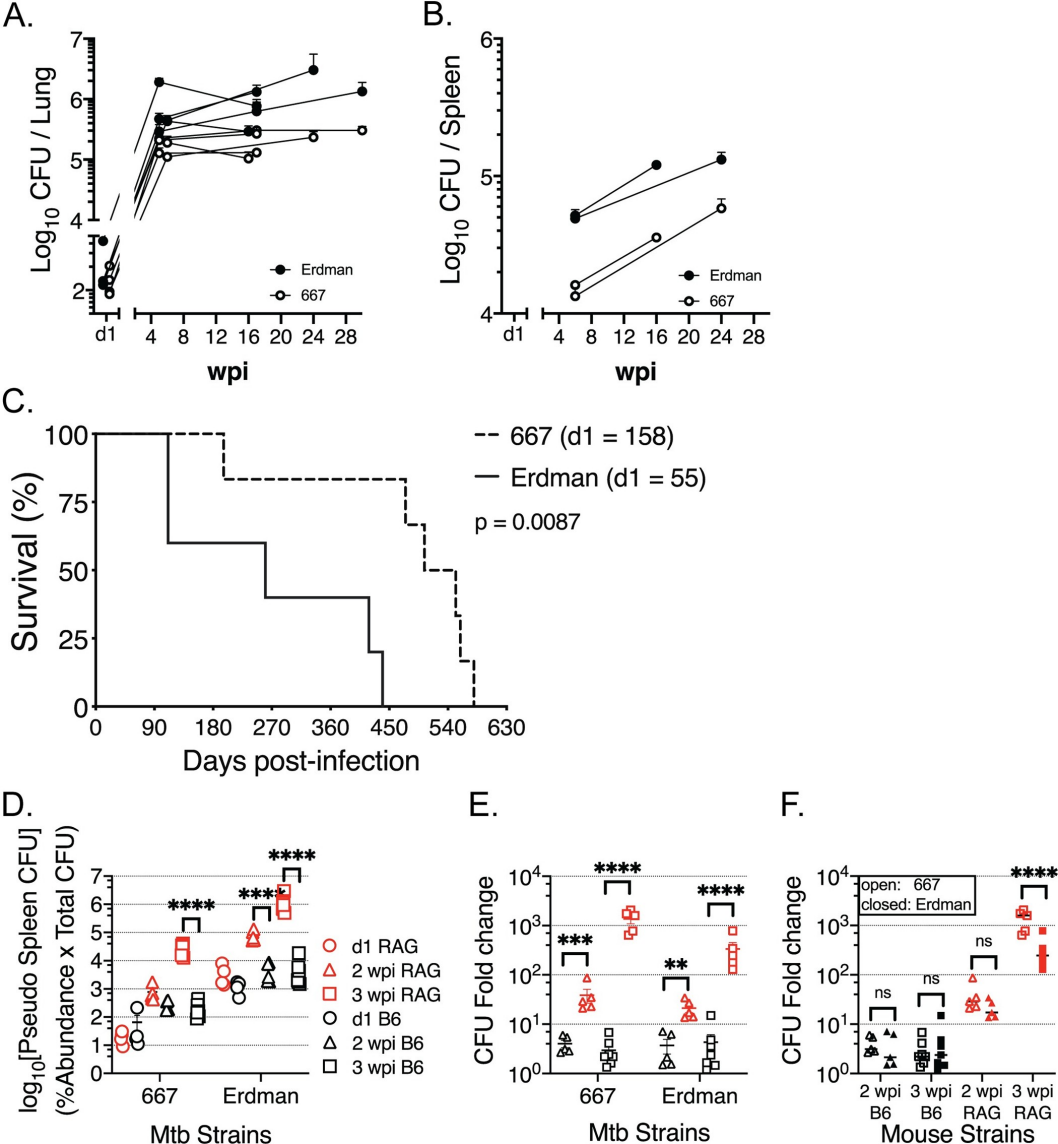
Infection with 667 is less virulent than Erdman. The bacterial burden is measured by CFU from lung (A) or spleen (B) homogenates from Erdman (filled) or 667 (open) infected C57BL/6J at different timepoints post infections. CFU data were compiled from 13 (lung) or 6 (spleen) independent experiments, from 4 to 30 weeks post infections. (C) The survival of C57BL/6 mice after Erdman (solid) or 667 (dashed) infection, which is one of two independent results with similar results. In this experiment, the d1 CFU was 158 (667) or 55 (Erdman). (D-E) A barcoded pool of clinical Mtb isolates was administered intravenously to C57BL/6J (black) or RAG1 KO (red) mice, and 4–8 mice of each strain were harvested for lungs and spleens to recover bacteria at 1 (circle), 14 (triangle), and 21 (square) days post infection. (D) The pseudo CFU of 667 and Erdman in spleen were determined by the relative abundance of the respective Mtb strains multiplied by total CFU in the spleen. (E) CFU fold-change (versus d1 CFU) of 667 or Erdman abundance in the spleen was compared between C57BL/6J (black) and RAG1 KO (red) mice. (F) CFU fold-change (versus d1 CFU) of 667 (open) vs. Erdman (closed) in the spleens of C57BL/6J (black) and RAG1 KO (red) mice. Statistical significance of survival curves (C) was determined by log-rank (Mantel-Cox) test; p value is shown. Statistical significance of CFU fold-change (D, E, F) were analyzed by a two-way ANOVA with Tukey (D) or Sidak’s (E, F) multiple comparison test. p values are indicated by asterisks: **, p<0.01, ***, p<0.001, ****, p<0.0001. Not all comparisons are shown for clarity.

We predicted that abolishing a “decoy antigen” would enhance the ability of the immune response to mediate protection against Mtb. To determine the ability of the immune response to control 667 vs. Erdman bacterial replication, mice were infected with each strain using a low-dose aerosol infection model. In 13 independent experiments across several time points, 667-infected mice had fewer CFU in the lung and spleen compared to Erdman-infected mice and had prolonged survival (Fig 3A–3C). Thus, it appears that 667 is less virulent than Erdman. However, as a clinical isolate, 667 is by definition virulent; and the Erdman strain used in these experiments has been passaged through mice to maintain its virulence. While the pathogenic potential of these two strains is determined in part by intrinsic factors (i.e., genetically determined), we wished to assess how differences in the adaptive immune response elicited by these two mycobacterial strains affects pathogenicity.

To determine whether the different T cell responses elicited by 667 and Erdman (see Fig 2) affected their growth *in vivo*, RAG1 KO and C57BL/6J mice were infected with a pool of barcoded Mtb clinical isolates, which also contained Erdman and 667, by the intravenous route. In the spleens of RAG1 KO mice, 667 grew similarly to Erdman, showing that in the absence of T and B cells, 667 did not have a growth defect (Fig 3D). In C57BL/6J mice, which have an intact immune system, both strains were similarly controlled, as indicated by no change in their relative abundance between 2 to 3 weeks after infection (Fig 3E). When the relative increase of 667 and Erdman were directly compared, there was no difference except in the spleens of RAG1 KO mice 3 wpi (Fig 3F). Thus, 667 is not intrinsically attenuated and we conclude that adaptive immunity is critical in controlling bacterial growth, most clearly observed by the change in relative abundance between RAG1 KO and C57BL/6J mice, two- and three-weeks post-infection.

### The A10T polymorphism alters the CD8 T cell response elicited by Mtb infection

Our experiments with 667 and Erdman show that naturally occurring polymorphisms between pathogenic Mtb strains can alter the hierarchy of antigens that CD8 T cells recognize and affect the outcome of infection. As there are 1,700 snps between Erdman and 667, we next sought to determine if EsxH^A10T^ was sufficient to alter the hierarchy of the CD8 T cells response elicited by Mtb. Oligo recombineering was used to change a single amino acid in the TB10.4_4−11_ epitope from IMYNYPAM (i.e., the Erdman “allele”) to IMYNYPTM (the 667 “allele”), which allowed us to generate isogenic Erdman strains that differed only at this position (S5A Fig). Hereafter, we will refer to these isogenic strains as Erd.EsxH^WT^ and Erd.EsxH^A10T^, respectively. Using unmanipulated Erdman strain as a reference for normalization, these isogenic strains had equivalent mRNA expression of the genes that encode immunodominant antigens including *esxH* (S5B Fig).

**Fig 4:**
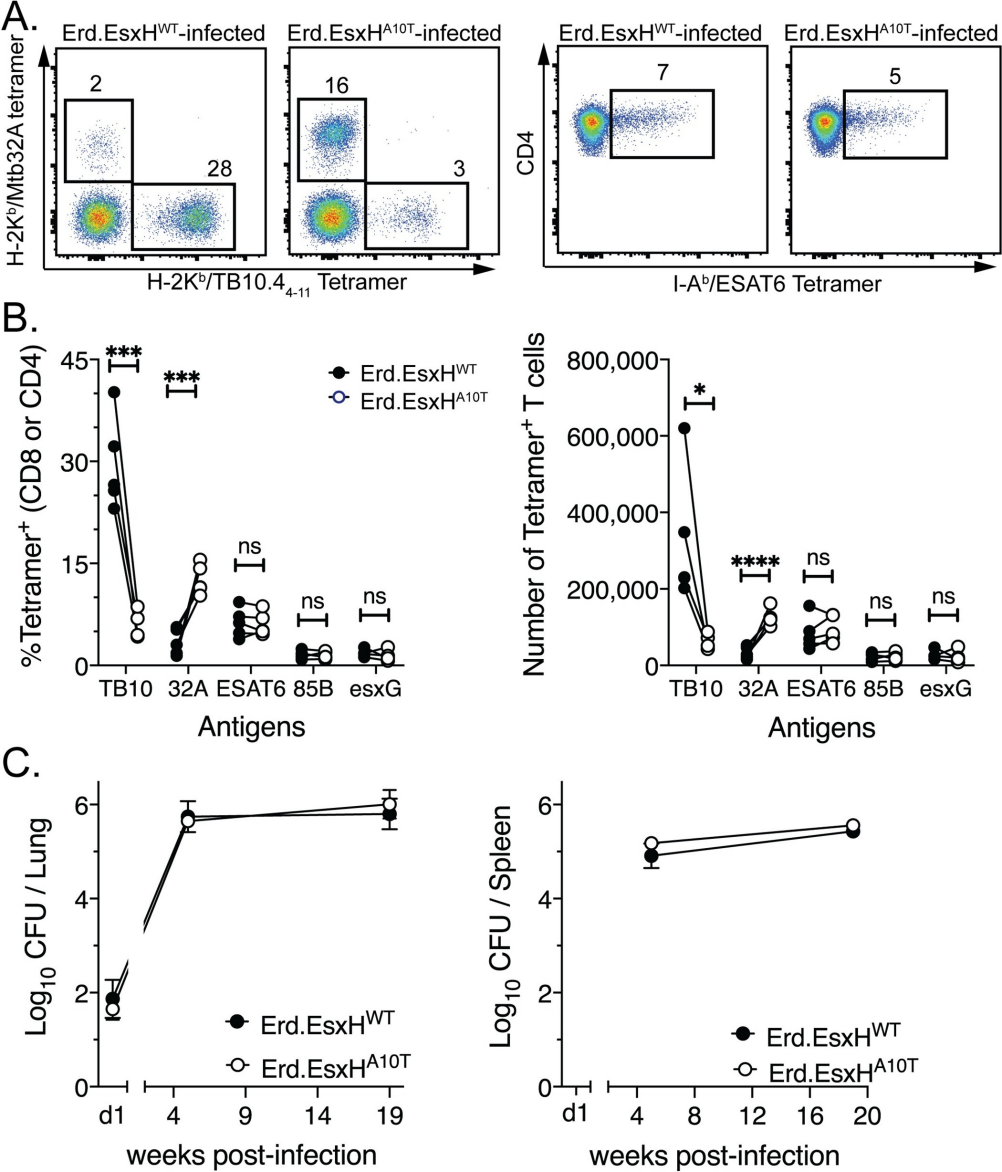
The A10T polymorphism leads to a change in immunodominance of the CD8 T cell response after 667 infection. C57BL/6J mice were infected with Erd.EsxH^A10T^ or Erd.EsxH^WT^ by the aerosol route and the lung T cell response was analyzed five weeks later by (A-B) tetramer staining and (C) the bacterial burden. (A) Representative flow cytometry plots of the frequency of MTB32a_309-318_- and TB10.4_4−11_-specific CD8 T cells (left), or ESAT6-specific CD4 T cells (right), after Erd.EsxH^WT^ or Erd.EsxH^A10T^ infection in the lungs of C57BL/6J mice. (B) The frequency and absolute number of antigen-specific T cells elicited by five independent paired infections of isogenic Erd.EsxH^WT^ (open) and Erd.EsxH^A10T^ (closed) strains determined by tetramer staining. The TB10-specific CD8 T cell responses elicited by the isogenic strains Erd.EsxH^WT^ and Erd.EsxH^A10T^, was determined using the specific tetramers ^WT^TB10.4_4−11_/K^b^ and ^A10T^TB10.4_4−11_/K^b^ tetramers, respectively. Each point is the average of 5 mice and lines connect the paired infections. (C) The bacterial burden was measured by CFU from lung (left) or spleen (right) homogenates from Erd.EsxH^WT^ or Erd.EsxH^A10T^ infected C57BL/6J at different timepoints post infections. CFU data were compiled from 4 independent experiments, from 5 to 19 weeks post infections. Statistical significance was determined by a two-tailed, unpaired Student’s T test. p values are indicated by asterisks: *, <0.05; **, p<0.01; ***, p<0.001; ****, p<0.0001.

We measured antigen-specific T cell responses elicited by infection with the isogenic strains Erd.EsxH^WT^ and Erd.EsxH^A10T^, using specific tetramers (i.e., the ^WT^TB10.4_4−11_/K^b^ and ^A10T^TB10.4_4−11_/K^b^ tetramers, respectively). After Erd.EsxH^A10T^ infection, there was a significant reduction in the TB10.4_4−11_-specific CD8 T cell response in C57BL/6J, as observed after 667 infection (Fig 4A and 4B). Similarly, the frequency and absolute number of MTB32a_309-318_-specific CD8 T cells was significantly increased after Erd.EsxH^A10T^ infection compared to Erd.EsxH^WT^ (Fig 4A and 4B). The CD4 T cell responses to ESAT6, Ag85B, and EsxG after Erd.EsxH^WT^ and Erd.EsxH^A10T^, were similar. Mtb-specific T cell response elicited by Erd.EsxH^WT^ or Erd.EsxH^A10T^ infection in BALB/c mice did not differ, similar to 667 infection (S6 Fig). When determined whether the change in immunodominance was associated with improved bacterial control, the bacterial burden in the lung and spleen did not significantly differ between Erd.EsxH^WT^ versus Erd.EsxH^A10T^ infected mice (Fig 4C). Thus, the EsxH^A10T^ polymorphism is sufficient to alter the hierarchy of immunodominant CD8 T cell responses in C57BL/6J mice, but under these conditions did not affect bacterial growth.

### 667-infected macrophages inefficiently present Mtb antigens to CD8 T cells

The CD8 T cell contribution to Mtb control can be difficult to demonstrate, especially in the face of an intact CD4 T cell response. Therefore, we next sought to determine whether altering the hierarchy of immunodominant CD8 T cell responses led to function changes in the recognition of infected macrophages. We previously reported that TB10.4_4−11_-specific CD8 T cells do not recognize infected macrophages [9]. Given the dominance of the TB10.4_4−11_-specific CD8 T cell response in the lung, we hypothesized that TB10.4 might be acting as a decoy antigen. Our finding that following 667 and Erd.EsxH^A10T^ infections, few TB10.4_4−11_-specific CD8 T cells were detected and instead, a greater expansion of MTB32a-specific CD8 T cells were elicited, supported this idea (Fig 2 and Fig 4). To further test this hypothesis, we determined whether abrogation of the immunodominant TB10.4_4−11_ epitope resulted in an expansion of CD8 T cells that better recognized Mtb-infected macrophages. Therefore, we quantified T cell recognition of Mtb-infected macrophages by the Mtb-infected macrophage intracellular cytokine staining assay (MIM-ICS) [30].

**Fig 5:**
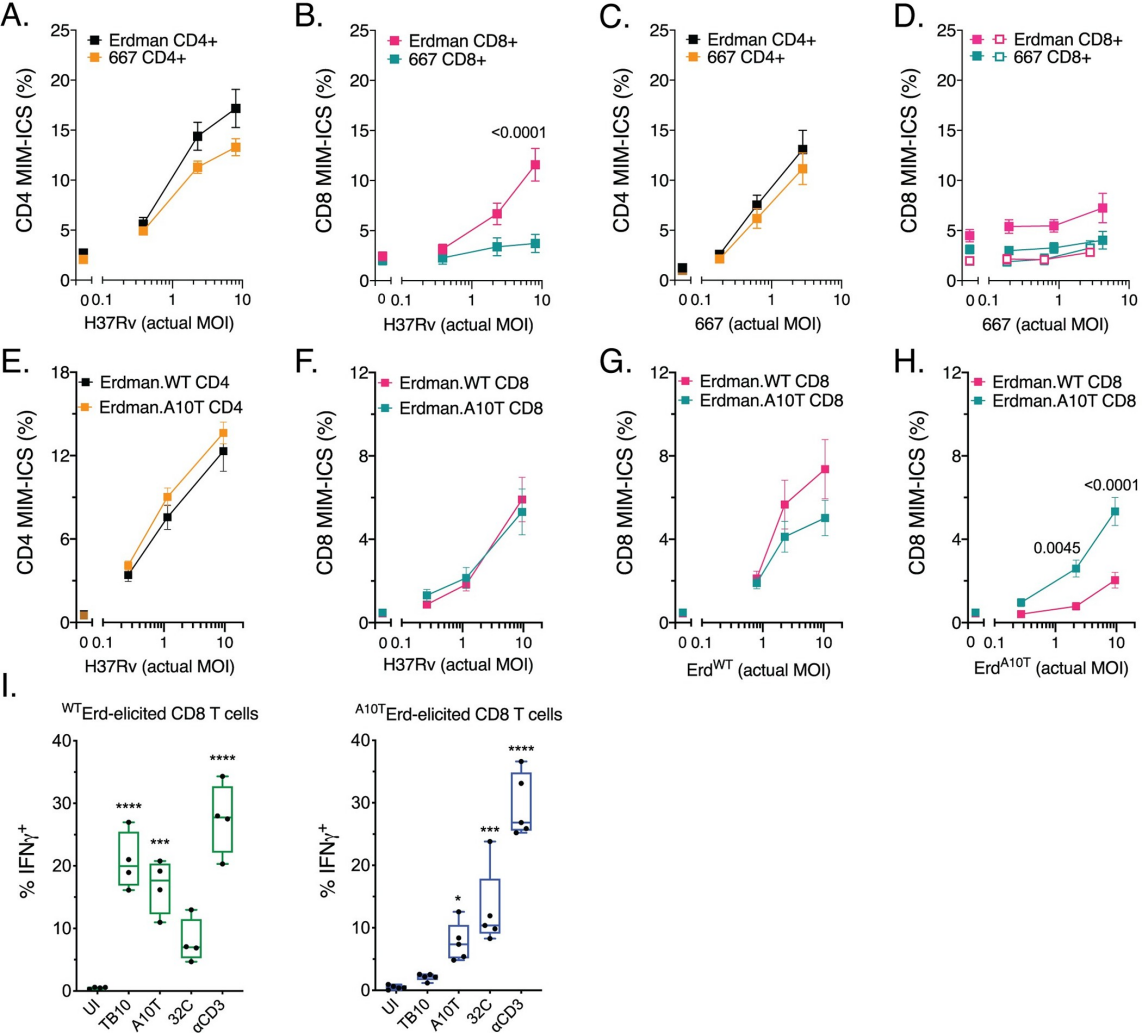
T cell recognition of infected macrophages. CD4 (A, C) or CD8 T cells (B, D) purified from the lungs of Erdman- or 667-infected mice were cultured with H37Rv-infected (A, B) or 667-infected (C, D) macrophages and the MIM-ICS assay was performed. MIM-ICS assay of isogenic strains elicited (E) CD4 T cells and (F) CD8 T cells in recognizing H37Rv-infected macrophages. Isogenic strains elicited CD8 T cells were also measured for their recognition ability of (G) Erd.EsxH^WT^-infected and (H) Erd.EsxH^A10T^-infected macrophages. The X axis is the actual multiplicity of infection (MOI) as determined by CFU plating. Statistical significance was determined by multiple t testing, and p values <0.05 are shown above the bars. (I) Erd.EsxH^WT^- or Erd.EsxH^A10T^-elicited CD8 T cells were cultured with uninfected macrophages (UI) or macrophages plus the indicated peptide epitopes or anti-CD3 mAb. Statistical significance was determined by one-way Anova, and p values <0.05 are shown above the bars. Results are representative of two (A-H) or three (I) different independent experiments.

Pulmonary T cells were purified from the Erdman- or 667-infected C57BL/6J mice and co-cultured with H37Rv-infected macrophages. IFNγ production was measured by the MIM-ICS assay as a readout of T cell recognition [30]. CD4 T cells from Erdman- or 667-infected C57BL/6J mice recognized H37Rv-infected macrophages similarly, and in a dose-dependent manner (Fig 5A). Thus, these two bacterial strains elicit CD4 T cell responses that had a similar capacity to recognize infected macrophages. There was little or no recognition of infected macrophages by CD8 T cells at a low MOI, as previously reported [30]. Contrary to our prediction, Erdman-elicited CD8 T cells, but not 667-elicited CD8 T cells, recognized H37Rv-infected macrophages at a high MOI (Fig 5B).

We considered whether the inability of 667-elicited CD8 T cells to recognize H37Rv-infected macrophages could reflect a strain- or lineage-specific CD8 T cell response. To address this question, we performed the reciprocal experiment and measured the ability of CD4 or CD8 T cells from Erdman- or 667-infected mice to recognize 667-infected macrophages. CD4 T cells from Erdman- and 667-infected mice recognized 667-infected macrophages similarly (Fig 5C). In contrast, neither Erdman nor 667-elicited CD8 T cells significantly recognized 667-infected macrophages (Fig 5D). When considering the bacterial loads of *in vitro* H37Rv and 667 infections, the actual MOIs were not significantly different among paired conditions (S7A Fig), suggesting that the bacterial uptake or intracellular growth were similar. These results show how the mycobacterial strain can profoundly affect antigen presentation and subsequent CD8 T cell responses.

We next determined whether the altered CD8 T cell response elicited by Erd.EsxH^A10T^ affected global recognition of Mtb-infected macrophages by T cells. CD4 and CD8 T cells from Erd.EsxH^WT^ and Erd.EsxH^A10T^ infected mice recognized H37Rv-infected macrophages similarly (Fig 5E and 5F). CD8 T cells from Erd.EsxH^WT^ and Erd.EsxH^A10T^-infected mice also recognized Erd.EsxH^WT^-infected macrophages similarly (Fig 5G). Since Erd.EsxH^A10T^ fails to elicit an immunodominant response to the TB10.4_4−11_ epitope, one may have expected Erd.EsxH^WT^ elicited CD8 T cells to recognize Mtb-infected macrophages better than CD8 T cells elicited by Erd.EsxH^A10T^. However, the similar recognition of Mtb-infected macrophages is consistent with the failure of the TB10.4_4−11_ epitope to be presented by Erd.EsxH^WT^ or H37Rv-infected macrophages [9]. These results were independently confirmed by showing that polyclonal CD8 T cells from Erdman-infected mice recognize *ΔesxH*- and *esxH*^*WT*^-complemented *ΔesxH*-infected macrophages similarly (S8 Fig). Surprisingly, Erd.EsxH^WT^-elicited CD8 T cells failed to recognize Erd.EsxH^A10T^-infected macrophages as well as Erd.EsxH^A10T^-elicited CD8 T cells (Fig 5H), despite the ability of antigen-specific CD8 T cells from both Erd.EsxH^WT^ and Erd.EsxH^A10T^ *in vivo* infections to activate and recognize their cognate epitopes (Fig 5I). Again, the bacterial loads of *in vitro* Erd.EsxH^WT^ and Erd.EsxH^A10T^ infections were not significantly different (S7B Fig). Thus, natural EsxH^A10T^ polymorphism induce distinct T cell responses that affect the ability of elicited T cells to recognize infected macrophages. We next sought to understand mechanistically why the EsxH^A10T^ polymorphism abrogated the CD8 T cell response to the TB10.4_4−11_ epitope.

### CD8 T cells recognize naturally occurring variants of the TB10.4_4−11_ epitope

**Fig 6:**
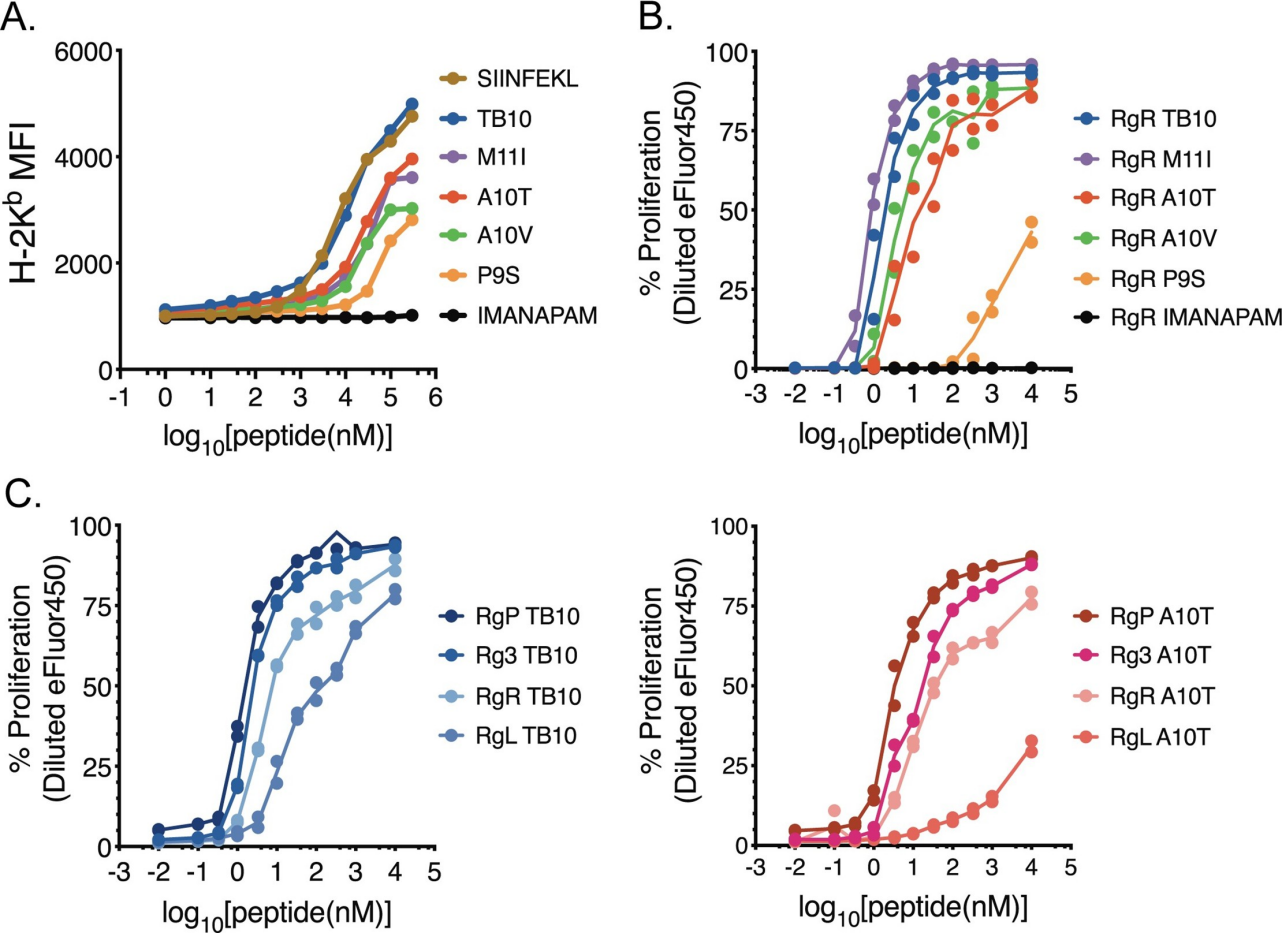
Naturally occurring variants of the TB10.4_4−11_ epitopes bind K^b^ and stimulate TB10.4_4−11_-specific CD8 T cells. (A) RMA-S K^b^ stabilization assay. Variant peptides were titrated, added to RMA-S cells, and the relative H-2 K^b^ surface expression (MFI) was determined by flow cytometry. The following peptides were used: SIINFEKL (positive control), IMANAPAM (negative control), “WT” TB10.4_4−11_, and the following variants of TB10.4_4−11_: A10T, A10V, P9S, and M11I. (B) The proliferation of the TB10.4_4−11_-specific CD8 T cell line TB10RgR was measured by eFluor450 dilution 48 hours after co-culture with macrophages pulsed with titrated amounts of the indicated peptides. (C) Proliferative response of the TB10RgP, TB10Rg3, TB10RgR, and TB10RgL CD8 T cell lines 48 hours after co-culture with macrophages pulsed with titrated amounts of WT TB10 peptide (left) or A10T peptide (right). Each assay was repeated 4 times.

The EsxH^A10T^ polymorphism could abrogate the CD8 T cell response because the epitope is no longer produced, because it fails to bind to class I MHC, or because it is no longer recognized by T cells. As the A10T polymorphism, similar to most *esxH* polymorphisms, is within the TB10.4_4−11_ epitope and doesn’t affect presentation of other TB10 epitopes (S4 Fig, S6 Fig), we hypothesized that the polymorphism would disrupt TB10.4_4−11_ binding to H-2 K^b^. Among the variant epitopes (i.e., P9S, A10T, A10V and M11I), only small differences were predicted in their binding to K^b^, compared to the WT dominant sequence (i.e., IMYNYPAM) and all had IC50 values between 5.6–9.5 nM (S3 Table). We experimentally confirmed these predictions by an RMA-S K^b^ stabilization assay using synthetic WT, P9S, A10T, A10V and M11I peptides (S2 Table). Data was analyzed after normalization and modeling using ECAnything (Prism 8). WT peptide stabilized K^b^ expression with an IC50 of 14,363, which was ~2-fold greater than the SIINFEKL peptide control (IC50: 7,318) (see Fig 6A, S9 Fig) [31]. The IMANAPAM peptide with Y6A/Y8A mutations, was designed and predicted to bind K^b^ 200-fold less than the WT epitope and failed to stabilize K^b^ (Fig 6A, S3 Table). The WT peptide was only 1.5-2-fold more effective at stabilizing K^b^ than A10T (IC50: 28,078), M11I (IC50: 29,075), A10V (IC50: 21,278), and 4.1-fold better than P9S (IC50: 59,349). Thus, it was unlikely that MHC binding could account for the inability of the A10T variant to elicit TB10.4_4−11_-specific CD8 T cells.

In the context of H2-K^b^, some of the polymorphic residues, including the 7^th^ amino acid of epitope, the location of the A10T substitution, were predicted to be solvent exposed and make contact with the TCR [31]. To address whether the variations in the TB10.4_4−11_ sequence affected T cell recognition, we used primary CD8 T cell lines elicited by *M*. *tuberculosis* Erdman and specific for the WT TB10.4_4−11_ epitope [9]. The TB10RgR CD8 T cell line recognized and proliferated in a dose-dependent manner to all polymorphic peptide epitopes with the following hierarchy WT ~ M11I > A10T ~ A10V > P9S (Fig 6B), an order similar to the peptide’s ability to stabilize cell surface K^b^ expression on RMA-S cells. Thus, we infer that T cell recognition was largely driven by peptide binding to K^b^. We used other TB10.4_4−11_-specific CD8 T cell lines (TB10Rg3, TB10RgL and TB10RgP), all with distinct TCRs. We compared the ability of TB10RgR, TB10Rg3, TB10RgL and TB10RgP to recognize the WT and A10T epitopes.

All four T cell lines recognized the two TB10 epitopes with a similar hierarchy: TB10RgP ~ TB10Rg3 > TB10RgR > TB10RgL (Fig 6C). While TB10RgP, TB10Rg3, and TB10RgR all recognized the WT and A10T epitopes similarly, TB10RgL only recognized the A10T epitope with low avidity. Importantly, the TCRs used by TB10RgR and TB10RgL differ by two amino acids–one in the CDR3α region and one in the CDR3β region [13]. As both of these T cells were elicited by the WT epitope, and were clonally expanded *in vivo* [13], these data show the fine specificity of TCRs for their cognate antigens, and reveals how T cells elicited against one Mtb strain (including vaccine strains) may be unable to recognize other Mtb strains. Importantly, these data show that the A10T epitope of 667 can both bind to K^b^ and be recognized by T cells. Thus, the different marked reduction of the TB10.4_4−11_-specific CD8 T cell response following 667 and Erd.EsxH^A10T^ infection cannot be explained by an inability of CD8 T cells to recognize the variant IMYNYPTM epitope.

### The A10T polymorphism alters TB10_4-11_ epitope production

Why the A10T substitution in the TB10.4 protein should abrogate the TB10.4_4−11_-specific CD8 T cell responses was unclear. As the A10T variant peptide (i.e., IMYNYPTM) could bind to K^b^ (Fig 6A, S3 Table) and was recognized by TB10.4_4−11_-specific CD8 T cells, we predicted Mtb strains with the A10T polymorphism should elicit TB10.4_4−11_-specific CD8 T cells. We next hypothesized whether the A10T polymorphism affects the antigen processing of the TB10.4 protein in Mtb-infected cells.

**Fig 7:**
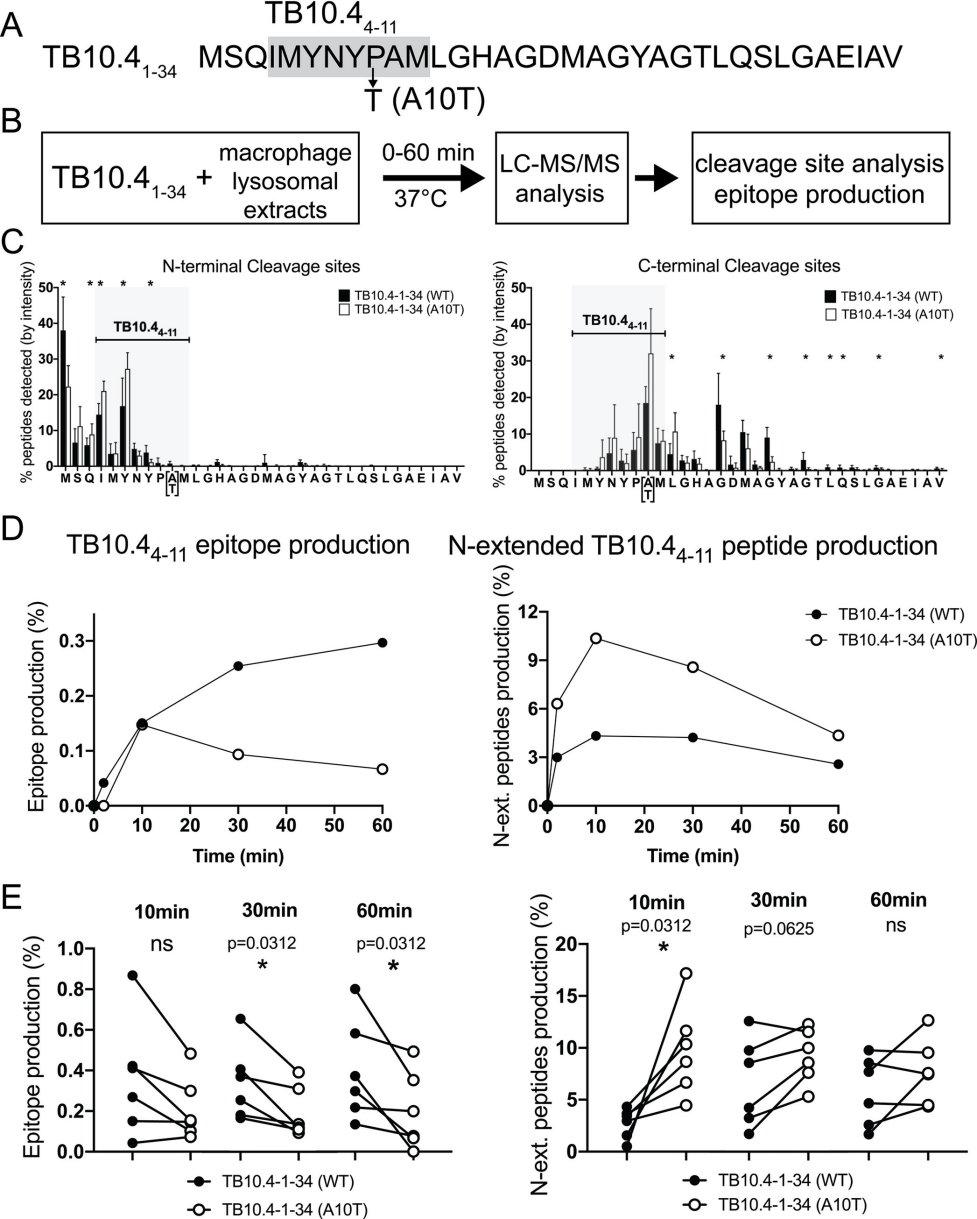
Different peptide degradation patterns of TB10.4_1−34_ with alanine or threonine at position 10 in macrophage lysosomes. (A) Amino acid sequence of 34-mer peptides (i.e., TB10.4_1−34_) containing alanine (WT) or threonine (A10T) at residue 10 (B) Experimental scheme of the *in vitro* peptide degradation assay in macrophage lysosomal extracts and WT and A10T TB10.4-1-34-mer sequences. (C) N- (left) and C- (right) terminus cleavage sites determined at 60 minutes. The relative amount of peptides starting (left panel) or ending (right panel) at each residue was quantified during the degradation of WT (black bars) and A10T (open bars) TB10.4-1-34-mer peptides. N = 6 experiments; *, p<0.05. (D) Production of TB10.4_4−11_ epitope (left panel) and N-extended TB10.4_4−11_ (right panel) from WT (black circles) or A10T 34-mer (open circles) at 60 minutes. One representative experiment. (E) Production of TB10.4_4−11_ epitope from WT (black circles) and A10T (open circles) TB10.4-1-34-mer peptides was determined at 10, 30, 60 minutes in six independent experiments. P values calculated with Wilcoxon matched-pairs signed rank test.

The processing of the TB10.4_4−11_ epitopes by lysosomal extracts from murine macrophages was quantified by mass spectrometry [31–33]. Briefly 34-mer peptides (i.e., TB10.4_1−34_) containing alanine (WT) or threonine (A10T) at residue 10 were incubated with C57BL/6J thioglycolate-elicited peritoneal macrophage (TG-PM) or bone-marrow derived dendritic cell (BMDC) lysosomal extracts and degradation peptides and cleavage sites were identified and quantified at various time points (Fig 7A and 7B, and S10A Fig). This assay recapitulates endogenous processing of epitopes in primary cells while allowing the analysis of peptide production and cleavage sites [31]. We analyzed the relative amount of peptides starting (N-terminus) or ending (C-terminus) at each residue during macrophage or BMDC lysosomal degradation (Fig 7C and S10B Fig). Both N- and C-terminal cleavage sites were mostly in the first third of the sequence. During macrophage lysosomal degradation, cutting sites after residues I4 and Y6 within epitope IMYNYP[A/T]M (TB10.4_4−11_) were significantly higher in the A10T 34-mer while Y8 (and Y21 outside the epitope) were more frequent in the WT sequence. In BMDC lysosomal extracts (from the same mice as the macrophages) enhanced N- and C-terminal cleavage sites were observed at residue Y2 within TB10.4_4−11_ epitope and at several sites outside the epitope (S10B Fig). These results are consistent with variant A10T altering peptide degradation patterns in macrophage and BMDC lysosomes.

We measured the production of TB10.4_4−11_ and N-extended TB10.4_4−11_ over a 1-hour degradation (Fig 7D; S10C Fig). The production of the TB10.4_4−11_ IMYNYPAM from the WT 34-mer was detected at 2 minutes only in macrophages (0.043% of total amount of peptides) and increased over 60 minutes (0.297%). In contrast the production of IMYNYPTM was slower (none detected after 2 minutes), peaked at 10 minutes (0.147%) and decreased (0.09 and 0.067% left at 30 and 60 minutes), leaving IMYNYPTM production 2.7 to 4.7-fold lower than IMYNYPAM production at these time points. In BMDC TB10.4_4−11_ IMYNYPAM and IMYNYPTM epitopes were not detected at 2 minutes but similarly produced at 10 and 30 minutes. The production of WT IMYNYPAM kept increasing to 0.499% while that of IMYNYPTM plateaued at 0.252% (S10C Fig left panel). Thus, in agreement with the degradation patterns, in both cell types, the production of IMYNYPTM is reduced due to higher sensitivity to lysosomal degradation. We also measured the production of N-extended TB10.4_4−11_ as N-extended peptides may still bind poorly to MHC but also reveal patterns of reduced antigen processing (Fig 7D and S10C Fig, right panels). In both macrophages and BMDC, IMYNYPTM peptides N-extended by up to 3aa were produced in higher quantity at all time points with a peak of at 10.36% at 10 minutes in macrophages and 4.9% in BMDC while N-extended IMYNYPAM remained <4.32% in macrophages and 2.5% in BMDC at all time points. These data suggest that IMYNYPTM extended by 1, 2, or 3 aa may not be efficiently trimmed, but possibly lead to shorter peptides that destroy the IMYNYPTM epitope, as seen by the presence of enhanced cleavage sites within IMYNYPTM. The higher production of IMYNYPAM compared to IMYNYPTM was also observed when the substrate was the first 25aa of the sequence showing the effect of the mutation on peptide production was not due to the use of a specific 34mer peptide.

We measured the production of TB10.4_4−11_ IMYNYPAM or IMYNYPTM and N-extended peptides in 6–7 different experiments with extracts from matched macrophages and BMDC from different batches of mice (Fig 7E, S10D Fig). While the amount of peptide produced in each experiment was variable between batches, the mutation A10T significantly reduced the production of IMYNYPTM peptide at 30–60 minutes in macrophages while its effect was less pronounced in BMDC. N-extended IMYNYPTM peptides were significantly better produced after a 30–60 minutes degradation in both cell types (Fig 7E, S10D Fig).

Altogether our data suggest that murine macrophages and to a lesser extent DC may not be able to process and present IMYNYPTM, or present it less efficiently, thus explaining the inefficient priming and expansion of TB10.4_4−11_-specific CD8 T cells following 667 or Erd.EsxH^A10T^ infection and the inefficient recognition of macrophages infected with 667. Therefore, the A10T polymorphism results in impaired processing of a CD8 T cell antigen and has a profound effect on the hierarchy of Mtb-specific CD8 T cells.

## Discussion

Here, we leverage a natural occurring polymorphism (i.e., the A10T substitution) in the EsxH protein to uncover the hierarchical structure of the antigen-specific CD8 T cell response elicited by Mtb. The TB10.4_4−11_-specific CD8 T cell response, which is immunodominant in C57BL/6J mice after H37Rv or Erdman infection, was largely absent after infection with 667 or Erd.EsxH^A10T^. This was accompanied by an expansion of the sub-dominant MTB32a_309-318_-specific CD8 T cell response. This outcome was surprising as the A10T and WT variants of the TB10.4_4−11_ epitope had similar abilities to bind K^b^ and stimulate T cell proliferation. Instead, a change in the processing of the TB10.4_4−11_ epitope modulated the CD8 response. To our knowledge, this is the first demonstration of a single amino acid substitution causing a major antigenic shift in the Mtb-specific CD8 T cell response following infection with virulent Mtb. These mechanistic insights provide a basis for reprograming host immune responses, which is an important consideration for the future design of vaccines against TB.

We previously reported that TB10.4_4−11_-specific CD8 T cells do not efficiently recognize infected macrophages [9]. We speculated that TB10.4 might act as a decoy antigen, which interferes with the expansion of CD8 T cells that recognize Mtb epitopes presented by Mtb-infected macrophages. To test this hypothesis, we engineered isogenic strain Erdman strains with the EsxH^A10T^ polymorphism, to determine how the overall CD8 T cell response would change in the absence of the TB10.4_4−11_ immunodominant epitope. The ability of polyclonal CD8 T cells, elicited in the presence or absence of the TB10.4_4−11_ epitope, to recognize Mtb-infected macrophages was measured by the MIM-ICS assay [30]. Two different aspects of CD8 responses were assessed by this assay: 1) the specificity and frequency of T cells elicited by Mtb *in vivo* (i.e., T cell priming); and 2) processing and presentation of Mtb antigens by infected macrophages *in vitro*. Varying the Mtb strain had no effect on CD4 T cell recognition of infected macrophages, indicating that our matching of the infectious dose of the different mycobacterial strains *in vivo* and *in vitro* was adequate. We found that 667-infected macrophages poorly stimulated CD8 T cells compared to H37Rv-infected macrophages, demonstrating antigen presentation can be altered by Mtb polymorphisms. Finally, Erd.EsxH^A10T^-elicited CD8 T cells recognized macrophages infected with Erd.EsxH^A10T^ better than with Erd.EsxH^WT^ (compare Fig 5G vs. 5H). From this result we infer that Erd.EsxH^A10T^ changes the repertoire of Mtb-specific CD8 T cell elicited by *in vivo* infection and alters class I MHC presentation by infected macrophages.

Our initial prediction was that Erd.EsxH^A10T^ infection would elicit a higher frequency of CD8 T cells capable of recognizing infected macrophages than Erd.EsxH^WT^. While both Erd.EsxH^A10T^- and Erd.EsxH^WT-^elicited CD8 T cells recognize H37Rv- and Erd.EsxH^WT^-infected macrophages similarly, only Erd.EsxH^A10T^-elicited CD8 T cells recognized Erd.EsxH^A10T^-infected macrophages. This loss of recognition by Erd.EsxH^WT-^elicited CD8 T cells instead of a gain of recognition by Erd.EsxH^A10T^-elicited CD8 T cells suggest a more complicated interpretation. There are likely to be other decoy antigens that become dominant when the response to TB10.4 is abrogated. A strong candidate is Mtb32a_309-318_. Despite the greater Mtb32a_309-318_-specific CD8 T cell response after Erd.EsxH^A10T^ infection, there is no greater recognition of infected macrophages. The discrepancy between the frequency of CD8 T cells that recognize Mtb32a_309-318_ vs. infected macrophages suggests that Mtb32a_309-318_ might be a decoy antigen. While Erd.EsxH^A10T^- and Erd.EsxH^WT-^elicited CD8 T cells recognize H37Rv- and Erd.EsxH^WT^-infected macrophages similarly, the repertoire of antigens recognized might be different. Such a difference could give rise to the discrepancy in the recognition of Erd.EsxH^A10T^-infected macrophages. In addition, we don’t know whether the CD8 T cells elicited by Erd.EsxH^WT^ and Erd.EsxH^A10T^ are similar. We measure their IFNγ response, but there could be other CD8 T cells that are recognizing infected macrophages but not making IFNγ [32]. Other possibilities include global effects on T cell priming or antigen presentation. Clearly, the mechanistic basis for these changes is more complex than we had originally envisioned.

We wished to understand why the A10T amino acid substitution abrogated the TB10.4-specific CD8 T cell response, particularly when the variant epitope could bind and be recognized by CD8 T cells. As EsxH inhibits class II MHC antigen presentation [33], we hypothesized that that the A10T polymorphism altered EsxH protein function and affected class I MHC presentation. While we did not formally address this hypothesis, we found that the A10T polymorphism altered the processing of epitope as revealed by *in vitro* degradation assays. Thus, the IMYNYPTM epitope was more rapidly degraded, which provides a mechanism for why, despite its potential to bind to K^b^ and be recognized by CD8 T cells, this variant epitope is largely non-immunogenic. These results provide an example of how the recognition of peptide-pulsed APC and Mtb-infected cells can be discordant.

We previously proposed that TB10.4 acts as a decoy antigen by eliciting an immunodominant CD8 T cell response, which cannot recognize infected macrophages [9]. Our new results are consistent with the immunodominance of the CD8 T cell response to TB10.4 arising by immunodomination. Immunodomination is the suppression of subdominant CD8 T cell responses by an immunodominant CD8 T cell response and develops because of competition for APC [16–21]. By ablating the immunogenicity of the TB10.4_4−11_ epitope, the CD8 T cell response to MTB32a becomes dominant, establishing that the TB10.4-specific CD8 T cell response diminishes the expansion of CD8 T cells specific to other Mtb antigens. The dramatically altered T cell response toward previously sub-dominant epitopes establishes that immunodomination shapes the CD8 T cell response to Mtb. A future area for investigation is to determine how competition for APC shapes the CD4 and CD8 T cell responses to Mtb.

A second prediction of our decoy hypothesis is that by eliminating the “decoy antigen,” other CD8 T cell responses would expand, recognize Mtb-infected macrophages, and mediate protection. The altered CD8 T cell response, greater bacterial control, and prolonged survival of mice infected with 667 compared to Erdman was consistent with this hypothesis. Proving that this difference was due to an altered CD8 T cell response could have pursued by infecting CD8-deficient mouse strains (e.g., CD8a KO). However, as there are ~1700 snps between 667 and Erdman, we focused on the isogenic Erdman strains. As C57BL/6 mice controlled Erd.EsxH^A10T^ and Erd.EsxH^WT^ infection similarly, the differences in bacterial burden and survival after Erdman and 667 infection cannot be attributed to the EsxH^A10T^ polymorphism. While there were no differences in CFU, the altered CD8 T cell response could have affected lung inflammation. Qualitatively, no differences in lung histopathology was observed 19 wpi, although quantitative criteria were not used to assess potential differences. We did not observe that reducing the TB10.4_4−11_ response resulted in better recognition of infected macrophages or increased protection *in vivo*. We do not yet know whether the MTB32a epitope is presented by Mtb-infected macrophages or whether the compensatory emergence of new dominant CD8 T cell responses also behaves as a decoy response. This possible redundancy in immunodominance and decoy antigens would not be surprising given the long evolutionary history of mycobacteria with the mammalian host, and the need for decoy antigens that would be presented by numerous HLA alleles.

Two distinct pressures affect the genetic evolution of human pathogens: 1) growth and replication; and 2) host immunity. The first leads to conservation of essential functional genes (e.g., metabolism and biosynthesis); the latter selects for microbes that can evade host immunity. Interestingly, many Mtb antigens that are recognized by human T cells are genetically conserved, implying that preserving T cell recognition of mycobacterial proteins contributes to bacterial fitness during human infection. Other mycobacterial genes that encode T cell antigens are polymorphic across different clinical isolates and lineages, and these may reflect an evolutionary response to immune pressure. Thus, the genetic heterogeneity among clinical Mtb isolates from TB patients could drive diverse T cell responses. The protein sequences of Mtb antigens used in vaccines are largely based on the H37Rv lab strain, and immune responses to vaccines and after infection, may be more heterogenous than previously expected. Ultimately, the A10T polymorphism, and maybe other SNPs in the *esxH* gene, acts as a CD8 T cell response regulator that shapes immunodominance by altering the function of TB10.4, through its interaction with vacuolar trafficking machinery [33], its regulation of metal ions [24] or its proteolysis (this manuscript). As *esxH* polymorphisms are focused in lineage 1 (L1), the geographical clustering of both Mtb L1 and MHC could be a manifestation of T cell pressure that promotes variation and ongoing immune evasion [34]. However, as genetic modification of an L4 lineage strain (i.e., Erd.EsxH^A10T^) was sufficient to the change the hierarchy of immune responses, the mechanism we describe should be relevant to all Mtb lineages.

Here we uncover a hierarchy of CD8 immune responses that appears to be established by immunodomination. We show that a single nucleotide polymorphism in the Mtb genome alters the CD8 T cell hierarchy in C57BL/6J mice and shifts the focus of the immunodominant response from TB10.4 to MTB32a. By focusing the CD8 T cell response on a decoy antigen such as TB10.4, Mtb accomplishes two things. First, the dominant CD8 T cell response is one that is unable to recognize Mtb-infected macrophages, and therefore, cannot mediate optimal protection. Second, the presumed presentation of TB10.4 by uninfected cells could promote inflammation and create an environment that promotes bacillary transmission [35, 36]. We find that the A10T polymorphisms alter the processing of the TB10.4_4−11_ epitope such that its abundance is diminished. Thus, not only is there less epitope available for T cell priming, but the effective abundance of Mtb peptide/MHCI complexes is likely to be below the activation threshold of CD8 T cells. These data are of great practical significance as they show how polymorphisms between circulating Mtb strains in a community, and BCG or H37Rv sequence-based vaccines could lead to a mismatch between the T cells that are primed by the vaccine and the epitopes presented by infected cells. Thus, there is uncertainty about whether antigens that are evolutionarily conserved versus those that are highly polymorphic should be incorporated into vaccines. Ultimately, an important goal is to identify the epitopes presented by Mtb-infected cells and to determine whether these Mtb antigens elicit protective T cells. This approach can provide a roadmap for rational vaccine design.

## Materials and methods

### Ethics statement

Studies were conducted using the relevant guidelines and regulations, and approved by the Institutional Animal Care and Use Committee at the University of Massachusetts Medical School (UMMS) (Animal Welfare A3306-01), using the recommendations from the Guide for the Care and Use of Laboratory Animals of the National Institutes of Health and the Office of Laboratory Animal Welfare.

### Animals

C57BL/6J, BALB/c and CB6F1 mice were purchased from Jackson Laboratories (Bar Harbor, ME) and housed under specific pathogen-free conditions at UMMS. Mice were 8 to 9 weeks old at the start of all experiments. Infected mice were housed in biosafety level 3 facilities under specific pathogen-free conditions at UMMS.

### Mycobacterium tuberculosis strains

Unless indicated, the Erdman strain was used for *in vivo* infections, and the H37Rv strain was used for *in vitro* infections. Erd.EsxH^WT^ and Erd.EsxH^A10T^ were generated as described below. The ΔesxH strain was a gift from Dr. Jennifer Philips (Washington University at St. Louis) [24, 33]. The barcoded clinical isolate pool was provided by Dr. Sarah Fortune (Harvard University) [27].

### Mouse infections

Eight- to nine-week old female mice were infected by the aerosol route. Frozen bacterial stocks were thawed, diluted in 0.9% NaCl with 0.02% Tween80, and sonicated before loading into a nebulizer for Glas-col aerosol chamber (Terre Haute, IN) to deliver approximately 100 CFU to the lungs of each mouse. The infecting dose was determined 16 hours after infection by plating lung homogenates on 7H11 agar plates (Hardy Diagnosis). Lungs and spleens were also aseptically removed, individually homogenized, and plated to determine viable bacteria.

### Preparation of cells

At different times post-infection, mice were euthanized, the lungs perfused with 10 mL of cold RPMI1640, and lung cell suspensions were prepared by coarse dissociation using the GentleMACS tissue dissociator (Miltenyi Biotec, Germany). Tissue was digested for 30 min at 37°C with 250 U/mL collagenase (Sigma) in RPMI1640 supplemented with 10 mM HEPES, 1 mM sodium pyruvate, 2 mM L-glutamine (all from Invitrogen Life Technologies, ThermoFisher, Waltham, MA) and 10% heat-inactivated fetal bovine serum (HyClone, GE Healthcare Life Sciences, Pittsburgh, PA). The tissue was further homogenized in the GentleMACS dissociator and sequential straining through 70 μm and 40 μm nylon cell strainers (Falcon). Pulmonary T cells were purified by positive selection using anti-CD90.2 microbeads on AutoMACS (Miltenyi Biotec, Germany). Thioglycolate-elicited peritoneal macrophages were obtained 4–5 days after intraperitoneal injection of donor mice with 3% thioglycolate solution [37]. CD11b microbeads (Miltenyi, Biotec, Germany) were used to purify the macrophages.

### Peptides

We used the Immune Epitope Database tools to predict differences between the variant TB10 peptide epitopes ability to bind H-2 K^b^ [38]. The WT (IMYNYPAM), negative control (IMANAPAM), Mtb32a_309-318_ (GAPINSATAM) and ESAT6_1-15_ (MTEQQWNFAGIEAAA) peptides were synthesized by New England Peptides (Gardner, MA). Variant peptides A10T, A10V, P9S, and M11I were synthesized by Genscript (Piscataway, NJ). The TB10.4 peptide library was obtained from BEI Resources.

### RMA-S assay

RMA-S cells were provided by Dr. Lawrence Stern laboratory (UMMS, Worcester MA). Titrated (100uM to 1pM) were added to RMA-S cells (5x10^4^/well) and cultured overnight at 27C in 5% CO_2_. The cells were the shifted to 37C for 1–2 hours, and then their surface expression of K^b^ was analyzed by flow cytometry.

### Measurement of T cell proliferation

Purified T cells were labeled with the cell proliferation dye eFluor450 (eBiosciences). Dye dilution was used as a measure of T cell proliferation, which was determined 72 hrs after co-culture with peptide-pulsed APC, by flow cytometry.

### In vitro infection

Frozen aliquots of Mtb were thawed, grown in 7H9 media until log-phase with an OD600 of 0.6–1, washed, opsonized with TB coat (RPMI 1640, 1% heat-inactivated FBS, 2% human serum, 0.05% Tween-80) and filtered (5μm) as previously described [9]. After manual counting, the bacteria were added to macrophages on UpCell Nunc plate (Thermo Fisher Scientific, MA) at the desired multiplicity of infection (MOI) and co-incubated overnight. The macrophages were subsequently washed and enumerated by trypan blue. The actual MOI was determined by lysing macrophages in replicate wells at final concentration of 1% (v/v) Triton X-100 and plating serial dilutions. CFU were enumerated after 21 days.

### Mtb-Infected Macrophage Intracellular Cytokine Staining (MIM-ICS)

The MIM-ICS assay was performed as described. Briefly, TGPMs were infected overnight, recovered, and plated (10^5^/well). 10 μM peptides and uninfected TGPMs were used as controls. Purified T cells from Mtb-infected mice were added and a standard ICS protocol was used [30].

### Peptide library screening

At the indicated timepoints, lungs and spleens were obtained from infected mice and single cell suspensions prepared. Each peptide in the library or control peptides (10 uM) were added to 10^5^ lung cells/well in triplicate. After 48 hours in 5% CO2 at 37C, supernatants were filtered (0.2 μm) and IFNγ measured by ELISA (Biolegend, CA).

### Flow cytometry

Samples were fixed with 1% paraformaldehyde in PBS for >1 hr before analysis with a MACSQuant flow cytometer (Miltenyi Biotec). FlowJo Software (Tree Star, Portland, OR) was used for data analysis. Single lymphocytes were gated by forward scatter versus height and side scatter for size and granularity, and dead cells were excluded. Cells were stained with Zombie Violet or Aqua Fixable viability dye, and the antibodies to: CD4 (GK1.5), CD8 (53–6.7), CD3ε (145-2C11), CD19 (6D5), CD44 (IM7), CD62L (MEL-14), CD127 (A7R34), KLRG1 (2F1/KLRG1), CD69 (H1.2F3), IFNγ (XMG1.2), F4/80 (BM8) and H-2 K^b^ (AF6-88.5) (all from Biolegend). Tetramers were obtained from the National Institutes of Health Tetramer Core Facility (Emory University Vaccine Center, Atlanta, GA).

### Barcoded clinical isolate pool infection

Barcoded Mtb strain generation, infection, and analysis were previously described [39]. Briefly, selected clinical isolates were individually tagged with unique 8 basepair sequence, grown to log phase, pooled, and used for intravenous infection at 1x10^6^ Mtb per mouse. At indicated time post infection, lungs and spleens were harvested, homogenized and plated on 7H10 supplemented with oleic albumin dextrose catalase (OADC) and 20 ng/ml kanamycin. After 3 weeks of incubation, the plates were counted for CFU, and colonies were scraped for genomic DNA extraction and sequencing by NextSeq and analysis using Python.

### Generation of isogenic Erd.EsxH^WT^ and Erd.EsxH^A10T^ by oligo recombineering

Oligo recombineering with long oligo containing desired base pair change were generated as described previously [40, 41]. Erdman was grown in 7H9 broth in log phase before electroporation of the pKM444 plasmid containing RecT annealase. Electroporated strains were selected on 7H10 agar plates containing 20 ng/ml kanamycin, and PCR used to verify the presence of pKM444 plasmid. An Erdman strain containing the Che9 phage RecT-producing plasmid was grown in 7H9 broth to an OD of 0.5. Anhydrotetracycline (Atc, final concentration, 500 ng/ml) was added to induce expression of RecT from the P_Tet_ promoter. The cells were grown overnight and prepared for electroporation with esxH^A10T^-conferring oligo (1 ug) and an oligo targeting the *rpsL* gene (0.1 ug) designed to generate a K43R mutation in the RpsL protein, which confers resistance to streptomycin. Following outgrowth, the culture was plated on 7H10 agar plates containing 20 ng/ml streptomycin. Selection of streptomycin screens for cells that pick up the DNA and are recombinogenic, increasing the frequency of finding the desired SNP. Candidate colonies were picked and screened for the targeted change by PCR analysis.

### In vitro epitope processing assay and mass spectrometry analysis

2 nmol of pure peptide (Biosynthesis, TX) was degraded with 15 ug of mouse macrophage or BMDC extracts at 37C in pH4 degradation buffer as described [42]. Aliquots were taken at various time points and the reaction was stopped by addition of 5% (v/v) of Formic acid (Thermo Fisher Scientific, MA) and the degradation products were purified by trifluoroacetic acid (Sigma-Aldrich, MO) precipitation (final concentration 5% (v/v)) and identified by in-house mass spectrometry as previously described [43]. Briefly, equal amounts of the purified degradation products were injected into a NanoLC Ultra-HPLC (Eksigent) for salt removal and separation, then online nanosprayed into an LTQ Orbitrap Discovery mass spectrometer (Thermo Fisher Scientific, MA) for identification. Peptides were separated in a NanoLC column (ChromXP C18, 3 um 120Å; Eksigent) over a gradient of 2–60% buffer B (buffer A: 0.1% (v/v) formic acid in MS-grade water (Fisher Scientific, NH); buffer B: 0.1% (v/v) formic acid in MS-grade acetonitrile (Fisher Scientific, NH) in 95 min with a conserved flow rate of 250 nl/min. Mass spectra were recorded in the 370–2000 Daltons range. In the tandem mass spectrometry mode, the eight most intense peaks were selected with a window of 1 Dalton and fragmented using helium as collision gas and a voltage of 35 V. Peaks in the mass spectra were searched against the source peptide databases with Proteome Discoverer (version 1.3; Thermo) and quantitatively analyzed. For a given peptide, the integrated area under the peak is proportional to the relative abundance of the peptide in the sample. Each sample was run on the mass spectrometer at least twice.

### Statistical analysis

Data are represented as mean ± standard error of the mean (SEM). For comparing two groups, A two-tailed, unpaired student’s t-test was used to compare two groups; a one-way ANOVA was used for >2 groups. A p value < 0.05 was considered significant. Analyses were performed using Prism (GraphPad Software, La Jolla, CA).

## Supporting information

S1 Fig The altered CD8 T cell hierarchy induced by 667 infection is stable over time. The total number of (A) TB10.4_4−11_-specific, or (B) MTB32a_309-318_-specific, CD8 T cells in the lungs of Erdman- or 667-infected mice detected using tetramers during the course of Mtb infection. Each point represents an individually analyzed mouse. (C) The proportion of tetramer-specific and other CD8 T cells that expressed KLRG1 or CD127. Closed bars, Erdman infection; open bars, 667 infection. Black, TB10-specific CD8 T cells; purple, MTB32a-specific CD8 T cells; teal, total CD8s after exclusion of TB10- and MTB32a-specific CD8s.
(PDF)

S2 Fig IMYNYPTM-loaded tetramers verify the loss of TB10.4_4−11_-specific CD8 T cells after 667 infection. (A) Competitive tetramer staining was performed using H-2K^b^/ ^WT^TB10.4_4−11_ and H-2K^b^/ ^A10T^TB10.4_4−11_ tetramers labelled with different fluorochromes in a single stain to assess the relative avidity of the responding CD8 T cells. CD8 T cells elicited by Erdman infection only bind the ^WT^TB10.4_4−11_/K^b^ tetramer indicating that their avidity for the WT epitope is greater than for the A10T variant. In contrast, CD8 T cells elicited by 667 bind better to the A10T tetramer but are also recognized by the WT tetramer. (B) Conventional tetramer staining of CD8 T cells elicited by Erdman (left) or 667 (right) infections, and stained with the H-2K^b^/ ^WT^TB10.4_4−11_ (top row) or H-2K^b^/ ^A10T^TB10.4_4−11_ (bottom row) tetramer, both in combination with the H-2K^b^/MTB32a_309-318_ tetramer (Y axis). Cells were gated by size, viability, and lymphocyte gate before finally gating on the CD8 T cell population.
(PDF)

S3 Fig No new TB10.4 epitopes were detected in the absence of the TB10.4_4−11_-specific CD8 T cell response. To detect whether any new epitopes of TB10.4 emerge following 667 infection, single cell lung suspensions from Erdman- or 667-infected (A) C57BL/6; (B) BALB/c; or (C) CB6F1 mice were incubated with peptides from a TB10.4 peptide library (21 peptides of 15mers overlapping 11 amino acids; also see S2 Table) and the indicated control peptides. Supernatants were collected after 48 hours and IFNγ production was detected by ELISA. Three strains of mice were used to identify whether the A10T polymorphism affects the production of T cells specific to TB10.4_4−11_ (K^b^ restricted; e.g., C57BL/6), TB10.4_20−28_ (K^d^ restricted) or TB10.4_74−88_ is (I-A^d^ restricted) (e.g., BALB/c). The F1 mouse was used to address whether any difference in the immunogenicity of TB10.4_4−11_ and TB10.4_20−28_ was modulated by host genetics. In this experiment, the 667-infected mice appear to have a greater IFNγ response induced by the ESAT-6 epitope. We observed variability in the ESAT6 response in these experiments but after several experiments concluded that these differences were not reproducible. The variability may depend on the type of assay used. For example, the number of ESAT6-specific tetramer+ cells in the lungs of Erdman and 667 infected mice is virtually indistinguishable (Fig 2D). The peptide screening used total lung cells (as opposed to purified T cells), which prevents us from normalizing the abundance of CD4 and CD8 T cells in each sample, which is routinely done in flow cytometry experiments by specifically gating on the T cells as is done in the flow experiments. Additionally, the exogenous peptides are not the only source of antigen as there are endogenous APC that are infected. Alternately, APC may secrete IL-12 and IL-18, which could drive antigen-independent IFNγ production. However, since this experiment is designed to identify new positive responses (e.g., cryptic epitopes); and not differences between the two strains, we believe that this experimental design is valid. Finally, another reason that the ESAT-6 induced IFNγ responses differs from the tetramer number is that the ESAT6/I-A^b^ tetramers may only stain a subset of the total ESAT6-specific T cells (e.g., high avidity)(for more discussion of this issue see Patankar et al, Mucosal Immunology 2019).
(PDF)

S4 Fig Similar CD8 T cell responses are elicited by 667 and Erdman infection in BALB/c mice. The frequency of K^d^-restricted CD8 T cells specific for TB10.4_20−28_ and I-A^d^-restricted CD4 T cells specific for TB10.4_74−88_ were measured using tetramers after Mtb infection. Infected BALB/c mice were analyzed at 5 weeks post-infection with ~100 aerosolized Erdman or 667. (A) Representative flow cytometry plots of TB10.4_74−88_-specific CD4 T cells (top row), or TB10.4_20−28_-specific and EspA_150-158_-specific CD8 T cells (bottom row) following Erdman (left) or 667 (right). The percentages (B) and the total cell numbers (C) of tetramer specific cells from the lungs of BALB/c mice. The similar CD4 T cell response to the TB10.4_74−88_ epitope after 667 or Erdman infection makes it unlikely that the failure of 667 to elicit TB10.4-specific CD8 T cells is because the polymorphic TB10.4 protein (i.e., A10T) is less stable or abundant. Similarly, Erdman elicited a comparable TB10.4_20−28_-specific CD8 T cell response in BALB/c mice to 667 infection. These data suggest that the changes in the immunogenicity of TB10.4_4−11_ is due to an epitope specific effect, and not a change that affects the global CD8 T cell response. Similarly, we examined an independent epitope recognized by CD8 T cells after Mtb infection and found that both 667 and Erdman elicited similar frequencies of EspA_150-158_-specific CD8 T cells. Thus, the CD8 T cell response elicited in BALB/c mice does not appear to be affected by the polymorphisms present in 667, based on quantification of the response to EspA_150-158_ or TB10.4_20−28_. These data are representative of three different independent experiments. Statistical testing performed using a one-way ANOVA. ns, non-significant.
(PDF)

S5 Fig Generation of Erd.EsxH^WT^ and Erd.EsxH^A10T^ isogenic strains. (A) Schematic representation of oligo recombineering method to generate isogenic strains. (B) RT-PCR was used to determine Mtb antigen gene expression by Erdman, the two isogenic strains created by recombineering, Erd.EsxH^WT^ and Erd.EsxH^A10T^, and the clinical isolate 667, all after growth in 7H9 media. Relative expression was measured in comparison to the 16S ribosomal RNA housekeeping gene and normalized to the non-genetically modified Erdman strain (i.e., Erdman).
(PDF)

S6 Fig Infection with the Erd.EsxH^WT^ and Erd.EsxH^A10T^ isogenic strains does not alter the antigen-specific CD4 and CD8 T cells response in BALB/c mice. Infected BALB/c mice were analyzed at 5 weeks post-infection with ~100 aerosolized Erd.EsxH^WT^ or Erd.EsxH^A10T^ isogenic strains. (A) Representative flow cytometry plots of TB10.4_20−28_-specific and EspA_150-158_-specific CD8 T cells (top row), or TB10.4_74−88_-specific and Ag85A-specific CD4 T cells (bottom row) following Erd.EsxH^WT^ (left) or Erd.EsxH^A10T^ (right). The percentages (B) and the total cell numbers (C) of tetramer specific cells from the lungs of BALB/c mice. Here, the CD8 T cell response elicited in BALB/c mice does not appear to be affected by the polymorphisms present in Erd.EsxH^A10T^, based on quantification of the response to EspA_150-158_ or TB10.4_20−28_. These data are representative of three independent experiments. None of the comparisons between 667 and Erdman infected mice were significantly different based on statistical analysis performed using a one-way ANOVA. ns, not significant.
(PDF)

S7 Fig 667 and Erdman, and Erd.EsxHWT and Erd.EsxH^A10T^ infect macrophages similarly. After monoinfection, bacterial loads were lower and survival was longer after 667 infection compared to Erdman in C57BL/6 mice. This raises the possibility that the uptake of 667 by macrophages differs from Erdman. Our competition experiment (Fig 3) shows that 667 and Erdman are similarly fit in the spleens of T cell deficient mouse (i.e., RAG ko), and both are controlled by adaptive immunity (i.e., in C57BL/6 mice). There did appear to be a difference in fitness in the lung that we did not explore. When performing *in vitro* macrophage infections, 667 and Erdman appeared to infect macrophages similarly (A). We determined the actual MOI after each *in vitro* infection by lysing infected macrophages and plating serial dilutions of the lysate. By this measure, the MOI of Erdman and 667 was very similar, and any differences were more likely due to differences in counting the bacteria before infection, rather than differences in uptake or intracellular growth. We performed similar experiments to compare Erd.EsxH^WT^ and Erd.EsxH^A10T^ and observed they also had a similar ability to infect macrophages (B). Statistical analysis by a paired t-test showed that the difference in MOI was not significant (A,B).
(PDF)

S8 Fig T cell recognition of macrophages infected with EsxH-deficient compared to EsxH-expressing Mtb strains. CD4 (left) or CD8 (right) T cells purified from the lungs of Erdman-infected mice were cultured with *ΔesxH*-infected (pink) or *esxH*^*WT*^-complemented *ΔesxH* (ie. *ΔesxH*::*WT)*-infected (teal) macrophages and the MIM-ICS assay was performed. The X axis is the actual multiplicity of infection (MOI) as determined by CFU plating. Results are representative of two different experiments. Each data point is an average result from 5 individual mice.
(PDF)

S9 Fig Peptide stabilization of K^b^ expressed by RMA-S cells. Normalized and fitted data from Fig 6A.using the ECanything nonlinear regression model (Prism 8) to fit the curves. By setting F = 50, the IC50 was determined for each data set. The points are the experimental data, the solid lines are the fitted line, and the dashed lines are the 95% confidence intervals. This experiment was repeated four times with similar results.
(PDF)

S10 Fig Different peptide degradation patterns of TB10.4_1−34_ with alanine or threonine at position 10 in BMDC extracts. (A) Experiment scheme of the *in vitro* peptide degradation assay in bone-marrow derived dendritic cells lysosomal extracts. (B) N (left) and C (right) terminus cleavage sites determined at 60 minutes. The relative amount of peptides starting (left) or ending (right) at each residue was quantified during the degradation of WT (closed) and A10T (open) TB10.4-1-34-mer peptides. N = 6 experiments; stars p<0.05. (C) Production of TB10.4_4−11_ epitope (left) and N-extended IM8 (right) from WT (closed) or A10T 34-mer (open) at 60 minutes. One representative experiment. (D) Production of TB10.4_4−11_ epitope from WT (closed) and A10T (open) TB10.4-1-34-mer peptides was determined at 10, 30, 60 minutes in N = 6 independent experiments. P values calculated with Wilcoxon matched-pairs signed rank test.
(PDF)

S1 Table Identification of *esxH* nonsynonymous polymorphisms among clinical isolates. The number of each polymorphism was indicated among different Mtb lineages with the number of independent occurrences of each polymorphism in parentheses.
(PDF)

S2 Table Peptide sequences used in this study. (PDF)

S3 Table Predicted processing efficiency and binding affinity to H2-K^b^ of polymorphic TB10.4_4−11_ epitopes. Bold letters indicate polymorphic substitution.
(PDF)
