# Supplementary figures and images for: A natural polymorphism of Mycobacterium tuberculosis in the esxH gene disrupts immunodomination by the TB10.4-specific CD8 T cell response
Source: PLoS Pathog. 2020 Oct 19;16(10):e1009000. doi: 10.1371/journal.ppat.1009000 (PMC7597557; doi:10.1371/journal.ppat.1009000)

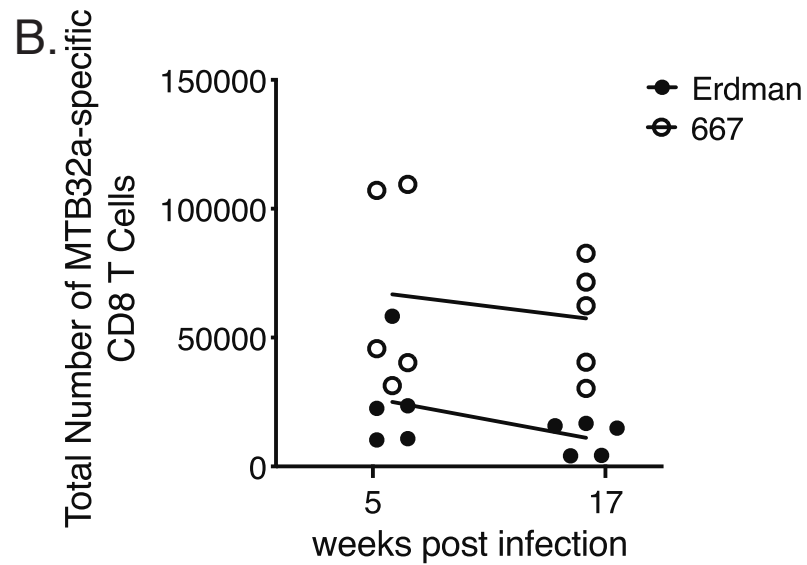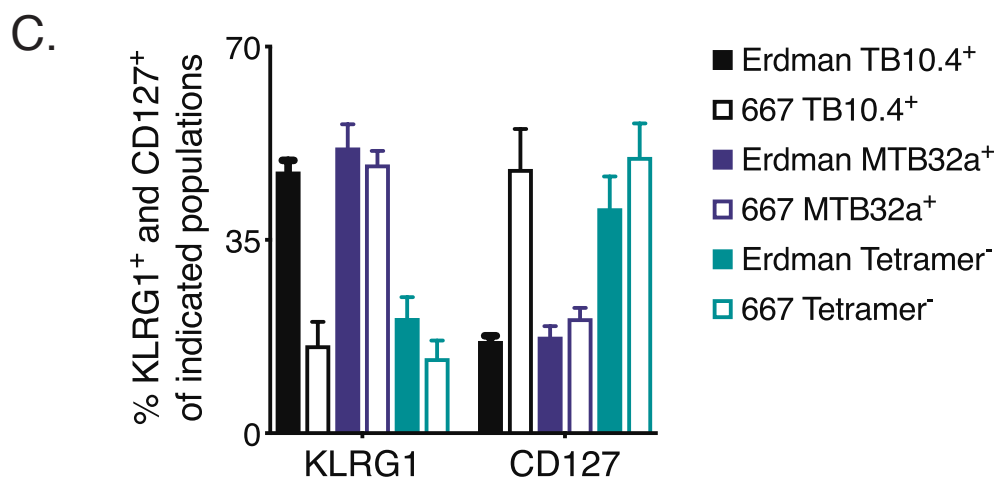

Supplement: S1 Fig — The total number of (A) TB10.44−11-specific, or (B) MTB32a309-318-specific, CD8 T cells in the lungs of Erdman- or 667-infected mice detected using tetramers during the course of Mtb infection. Each point represents an individually analyzed mouse. (C) The proportion of tetramer-specific and other CD8 T cells that expressed KLRG1 or CD127. Closed bars, Erdman infection; open bars, 667 infection. Black, TB10-specific CD8 T cells; purple, MTB32a-specific CD8 T cells; teal, total CD8s after exclusion of TB10- and MTB32a-specific CD8s. (PDF) [file ppat.1009000.s001.pdf]

A.

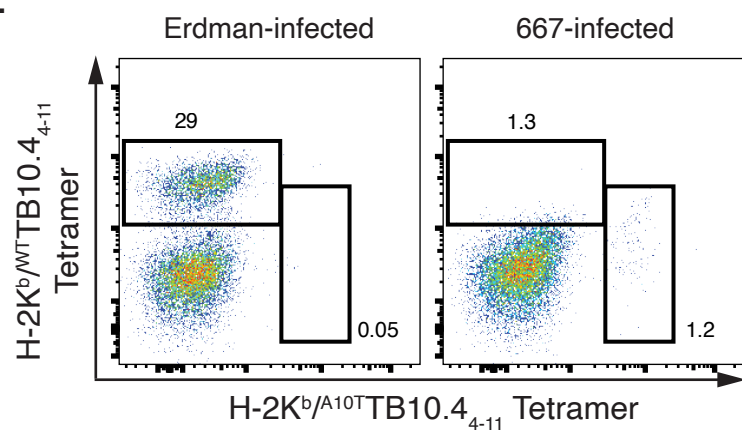

B.

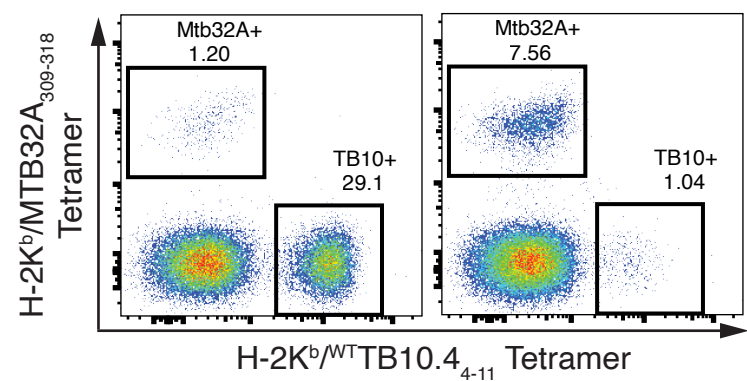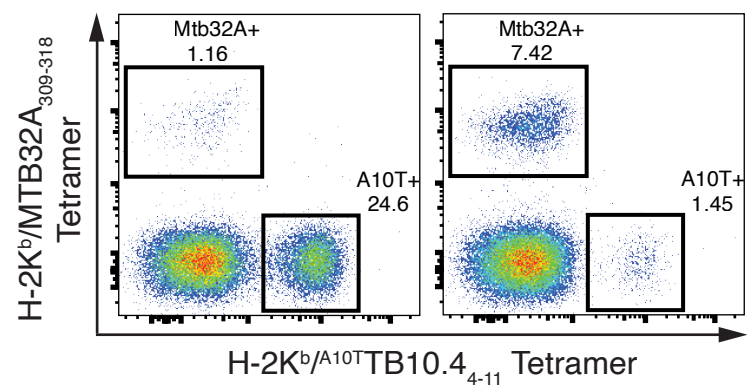

Supplement: S2 Fig — (A) Competitive tetramer staining was performed using H-2Kb/ WTTB10.44−11 and H-2Kb/ A10TTB10.44−11 tetramers labelled with different fluorochromes in a single stain to assess the relative avidity of the responding CD8 T cells. CD8 T cells elicited by Erdman infection only bind the WTTB10.44−11/Kb tetramer indicating that their avidity for the WT epitope is greater than for the A10T variant. In contrast, CD8 T cells elicited by 667 bind better to the A10T tetramer but are also recognized by the WT tetramer. (B) Conventional tetramer staining of CD8 T cells elicited by Erdman (left) or 667 (right) infections, and stained with the H-2Kb/ WTTB10.44−11 (top row) or H-2Kb/ A10TTB10.44−11 (bottom row) tetramer, both in combination with the H-2Kb/MTB32a309-318 tetramer (Y axis). Cells were gated by size, viability, and lymphocyte gate before finally gating on the CD8 T cell population. (PDF) [file ppat.1009000.s002.pdf]

A.

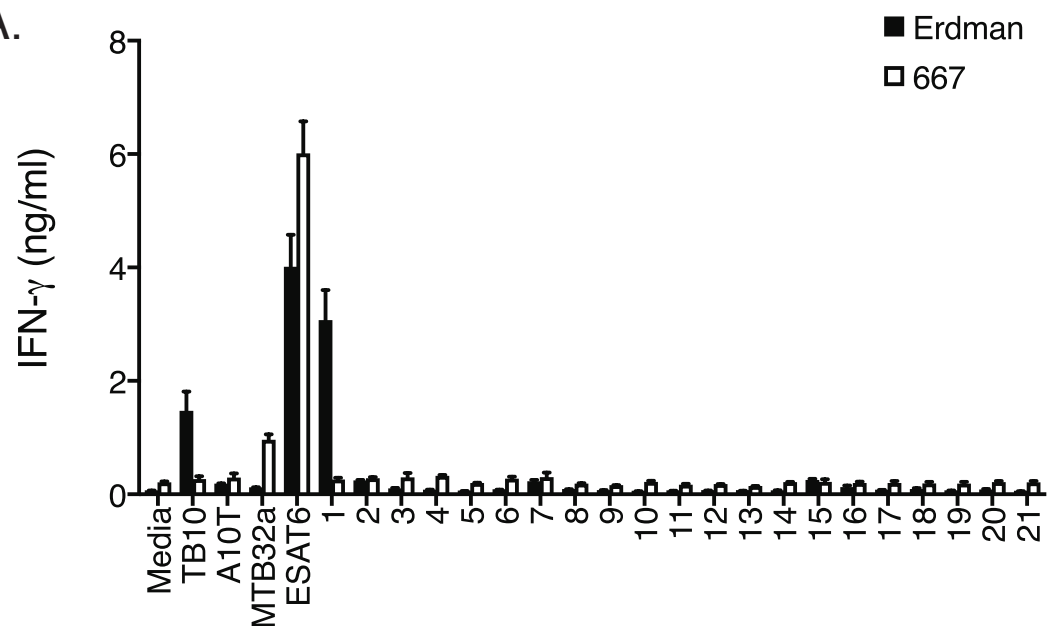

B.

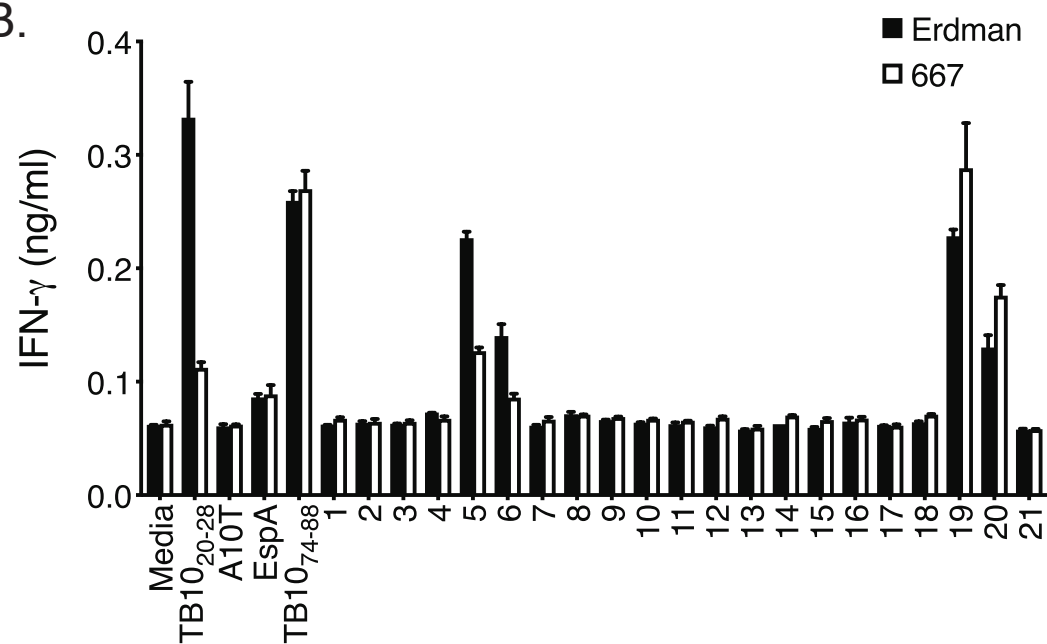

C.

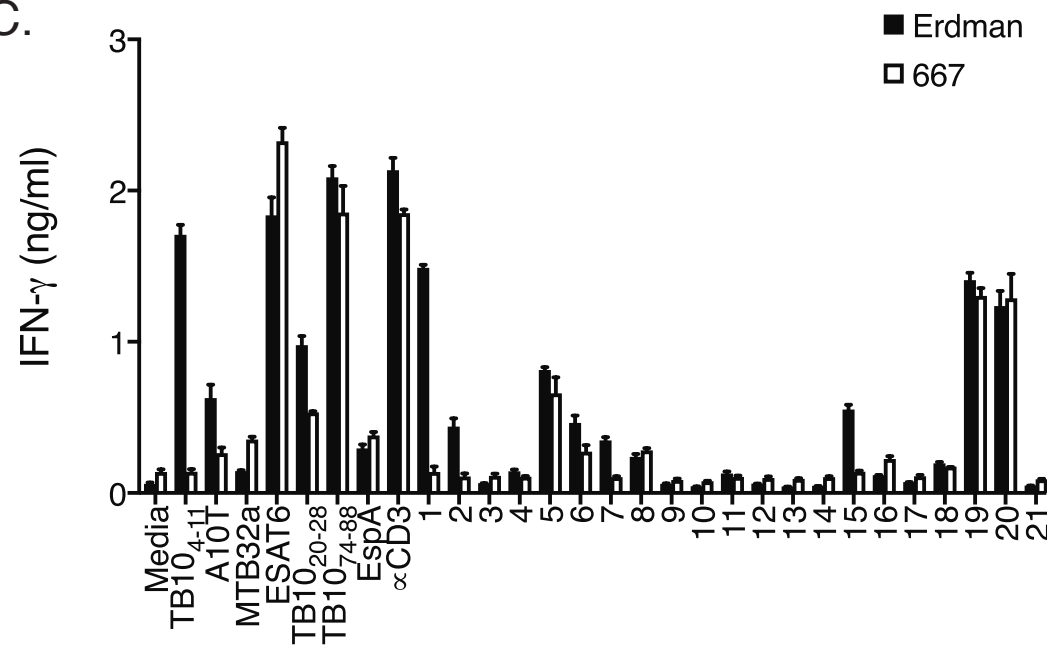

Supplement: S3 Fig — To detect whether any new epitopes of TB10.4 emerge following 667 infection, single cell lung suspensions from Erdman- or 667-infected (A) C57BL/6; (B) BALB/c; or (C) CB6F1 mice were incubated with peptides from a TB10.4 peptide library (21 peptides of 15mers overlapping 11 amino acids; also see S2 Table) and the indicated control peptides. Supernatants were collected after 48 hours and IFNγ production was detected by ELISA. Three strains of mice were used to identify whether the A10T polymorphism affects the production of T cells specific to TB10.44−11 (Kb restricted; e.g., C57BL/6), TB10.420−28 (Kd restricted) or TB10.474−88 is (I-Ad restricted) (e.g., BALB/c). The F1 mouse was used to address whether any difference in the immunogenicity of TB10.44−11 and TB10.420−28 was modulated by host genetics. In this experiment, the 667-infected mice appear to have a greater IFNγ response induced by the ESAT-6 epitope. We observed variability in the ESAT6 response in these experiments but after several experiments concluded that these differences were not reproducible. The variability may depend on the type of assay used. For example, the number of ESAT6-specific tetramer+ cells in the lungs of Erdman and 667 infected mice is virtually indistinguishable (Fig 2D). The peptide screening used total lung cells (as opposed to purified T cells), which prevents us from normalizing the abundance of CD4 and CD8 T cells in each sample, which is routinely done in flow cytometry experiments by specifically gating on the T cells as is done in the flow experiments. Additionally, the exogenous peptides are not the only source of antigen as there are endogenous APC that are infected. Alternately, APC may secrete IL-12 and IL-18, which could drive antigen-independent IFNγ production. However, since this experiment is designed to identify new positive responses (e.g., cryptic epitopes); and not differences between the two strains, we believe that this experimental design is valid. Finally, an [file ppat.1009000.s003.pdf]

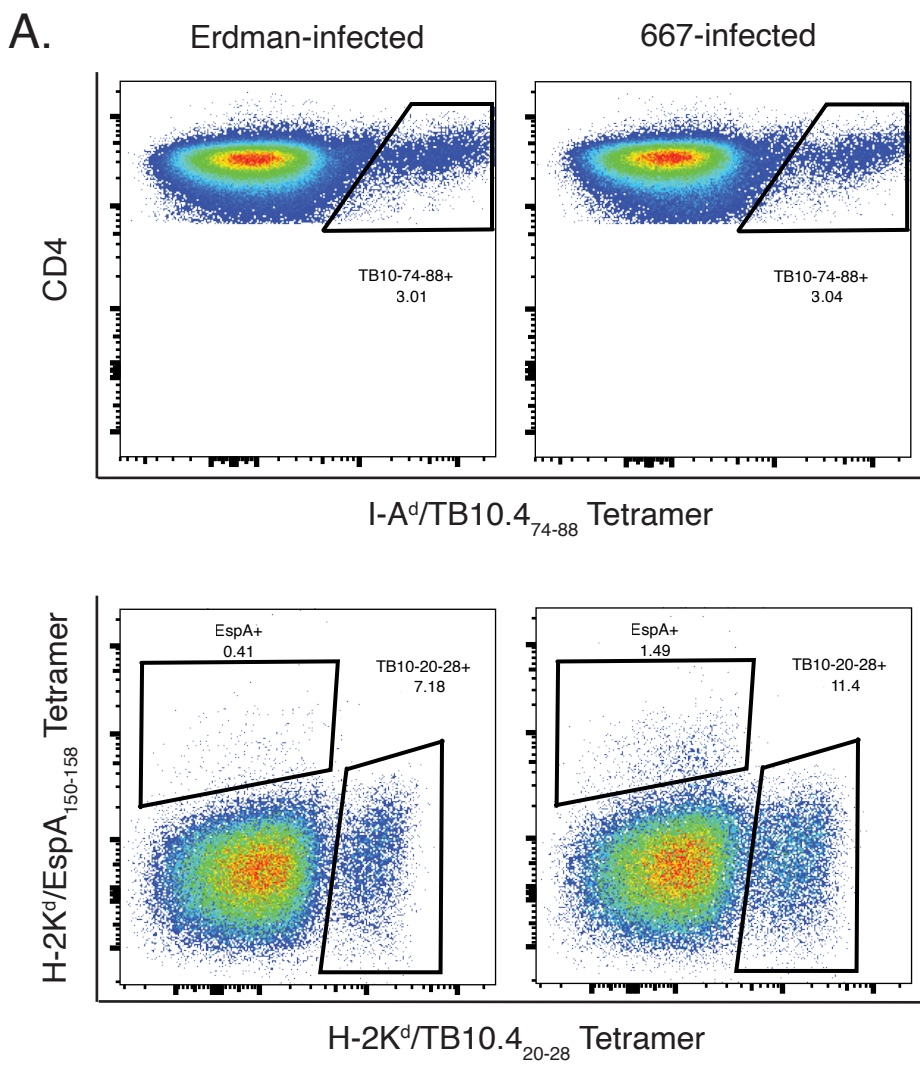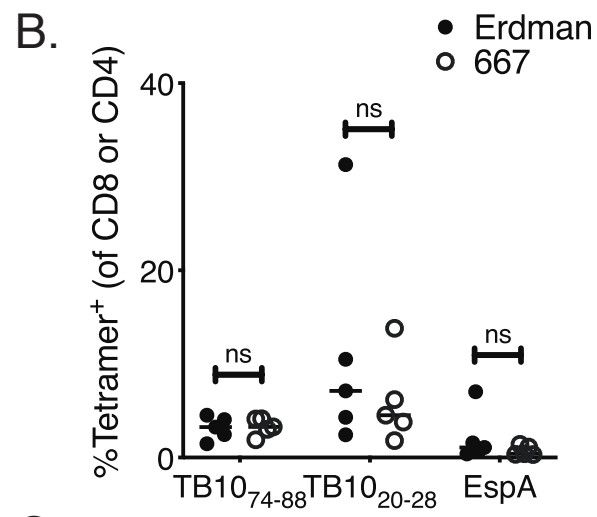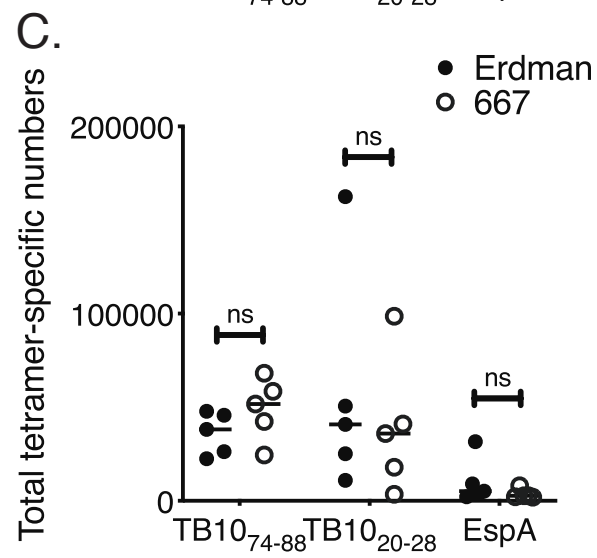

Supplement: S4 Fig — The frequency of Kd-restricted CD8 T cells specific for TB10.420−28 and I-Ad-restricted CD4 T cells specific for TB10.474−88 were measured using tetramers after Mtb infection. Infected BALB/c mice were analyzed at 5 weeks post-infection with ~100 aerosolized Erdman or 667. (A) Representative flow cytometry plots of TB10.474−88-specific CD4 T cells (top row), or TB10.420−28-specific and EspA150-158-specific CD8 T cells (bottom row) following Erdman (left) or 667 (right). The percentages (B) and the total cell numbers (C) of tetramer specific cells from the lungs of BALB/c mice. The similar CD4 T cell response to the TB10.474−88 epitope after 667 or Erdman infection makes it unlikely that the failure of 667 to elicit TB10.4-specific CD8 T cells is because the polymorphic TB10.4 protein (i.e., A10T) is less stable or abundant. Similarly, Erdman elicited a comparable TB10.420−28-specific CD8 T cell response in BALB/c mice to 667 infection. These data suggest that the changes in the immunogenicity of TB10.44−11 is due to an epitope specific effect, and not a change that affects the global CD8 T cell response. Similarly, we examined an independent epitope recognized by CD8 T cells after Mtb infection and found that both 667 and Erdman elicited similar frequencies of EspA150-158-specific CD8 T cells. Thus, the CD8 T cell response elicited in BALB/c mice does not appear to be affected by the polymorphisms present in 667, based on quantification of the response to EspA150-158 or TB10.420−28. These data are representative of three different independent experiments. Statistical testing performed using a one-way ANOVA. ns, non-significant. (PDF) [file ppat.1009000.s004.pdf]

A.

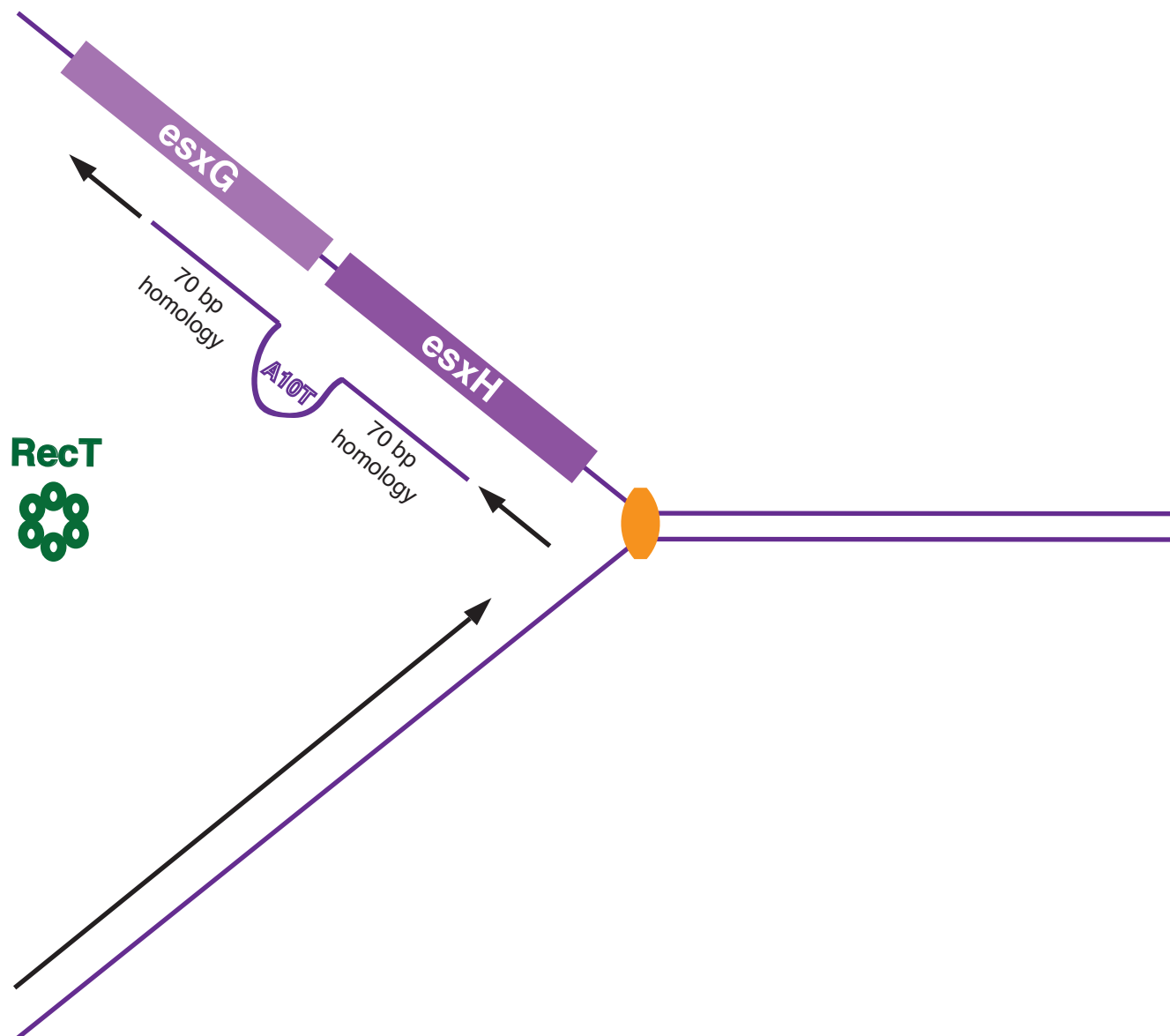

B.

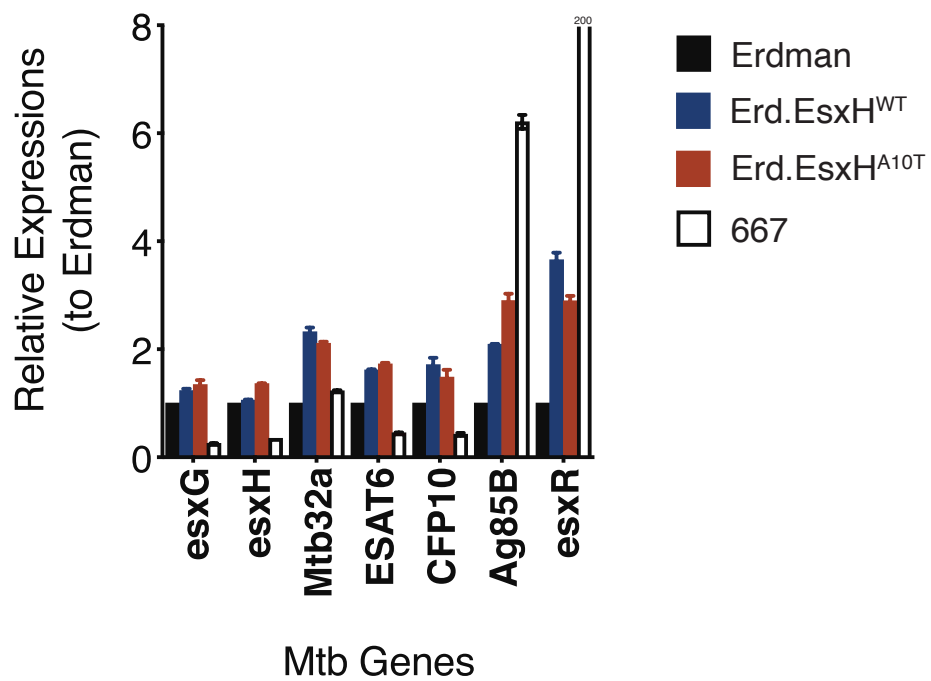

Supplement: S5 Fig — (A) Schematic representation of oligo recombineering method to generate isogenic strains. (B) RT-PCR was used to determine Mtb antigen gene expression by Erdman, the two isogenic strains created by recombineering, Erd.EsxHWT and Erd.EsxHA10T, and the clinical isolate 667, all after growth in 7H9 media. Relative expression was measured in comparison to the 16S ribosomal RNA housekeeping gene and normalized to the non-genetically modified Erdman strain (i.e., Erdman). (PDF) [file ppat.1009000.s005.pdf]

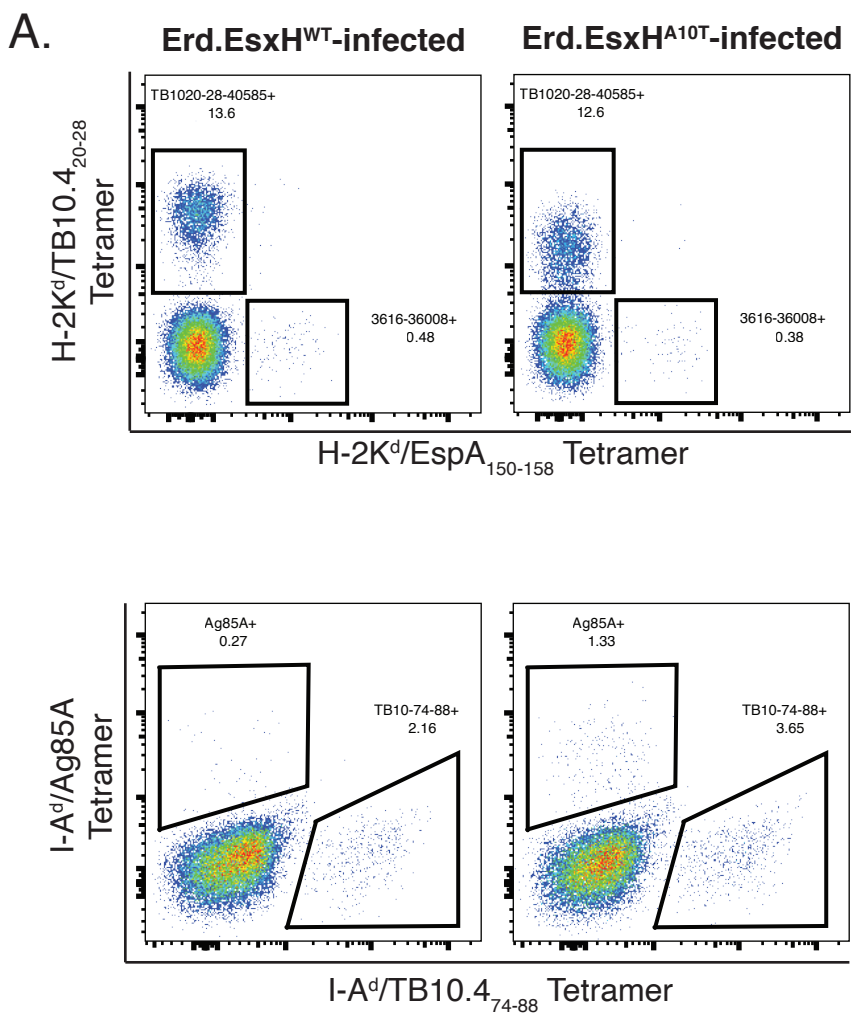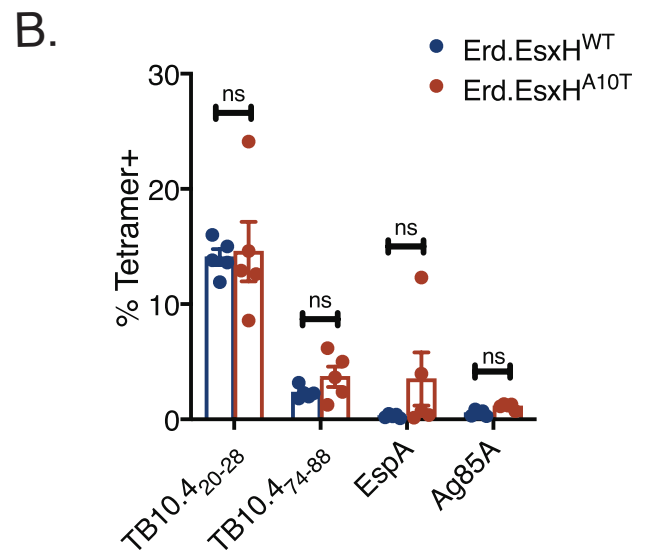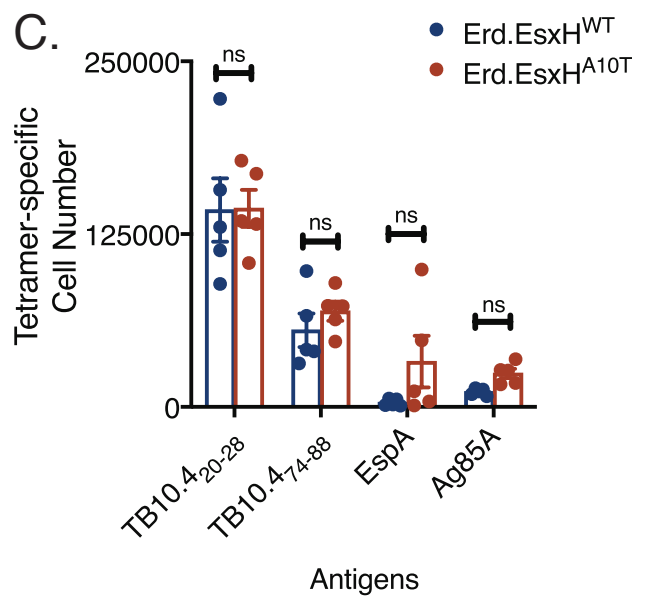

Supplement: S6 Fig — Infected BALB/c mice were analyzed at 5 weeks post-infection with ~100 aerosolized Erd.EsxHWT or Erd.EsxHA10T isogenic strains. (A) Representative flow cytometry plots of TB10.420−28-specific and EspA150-158-specific CD8 T cells (top row), or TB10.474−88-specific and Ag85A-specific CD4 T cells (bottom row) following Erd.EsxHWT (left) or Erd.EsxHA10T (right). The percentages (B) and the total cell numbers (C) of tetramer specific cells from the lungs of BALB/c mice. Here, the CD8 T cell response elicited in BALB/c mice does not appear to be affected by the polymorphisms present in Erd.EsxHA10T, based on quantification of the response to EspA150-158 or TB10.420−28. These data are representative of three independent experiments. None of the comparisons between 667 and Erdman infected mice were significantly different based on statistical analysis performed using a one-way ANOVA. ns, not significant. (PDF) [file ppat.1009000.s006.pdf]

A.

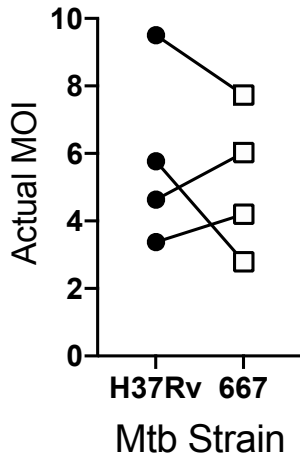

B.

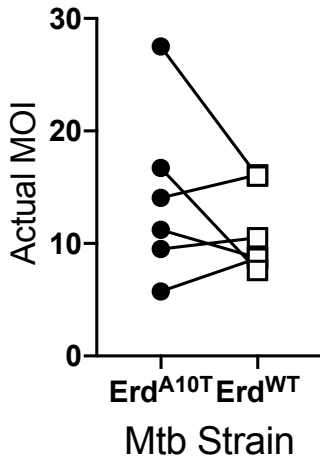

Supplement: S7 Fig — After monoinfection, bacterial loads were lower and survival was longer after 667 infection compared to Erdman in C57BL/6 mice. This raises the possibility that the uptake of 667 by macrophages differs from Erdman. Our competition experiment (Fig 3) shows that 667 and Erdman are similarly fit in the spleens of T cell deficient mouse (i.e., RAG ko), and both are controlled by adaptive immunity (i.e., in C57BL/6 mice). There did appear to be a difference in fitness in the lung that we did not explore. When performing in vitro macrophage infections, 667 and Erdman appeared to infect macrophages similarly (A). We determined the actual MOI after each in vitro infection by lysing infected macrophages and plating serial dilutions of the lysate. By this measure, the MOI of Erdman and 667 was very similar, and any differences were more likely due to differences in counting the bacteria before infection, rather than differences in uptake or intracellular growth. We performed similar experiments to compare Erd.EsxHWT and Erd.EsxHA10T and observed they also had a similar ability to infect macrophages (B). Statistical analysis by a paired t-test showed that the difference in MOI was not significant (A,B). (PDF) [file ppat.1009000.s007.pdf]

### CD4 T cells

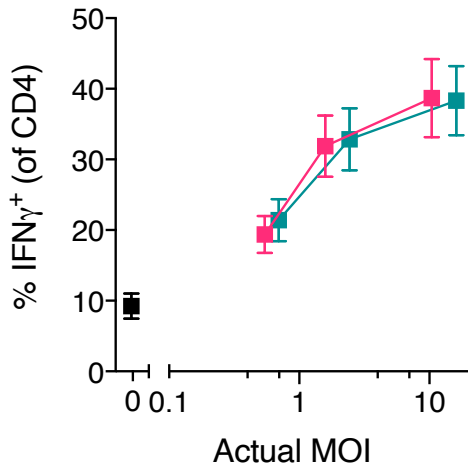

### CD8 T cells

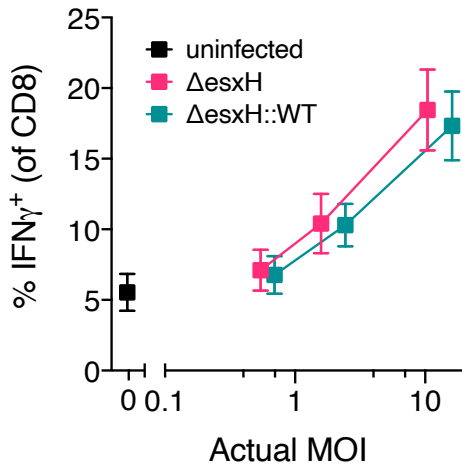

Supplement: S8 Fig — CD4 (left) or CD8 (right) T cells purified from the lungs of Erdman-infected mice were cultured with ΔesxH-infected (pink) or esxHWT-complemented ΔesxH (ie. ΔesxH::WT)-infected (teal) macrophages and the MIM-ICS assay was performed. The X axis is the actual multiplicity of infection (MOI) as determined by CFU plating. Results are representative of two different experiments. Each data point is an average result from 5 individual mice. (PDF) [file ppat.1009000.s008.pdf]

A

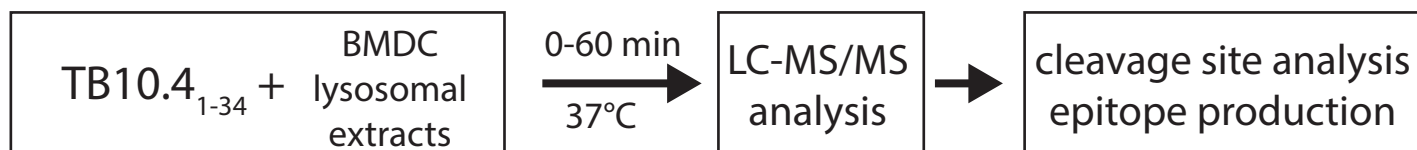

B

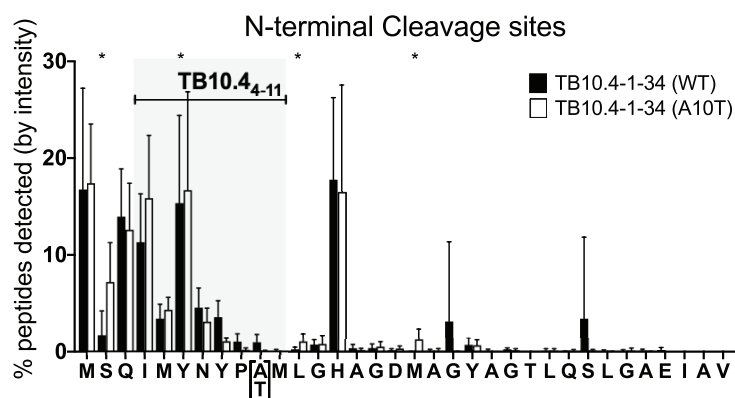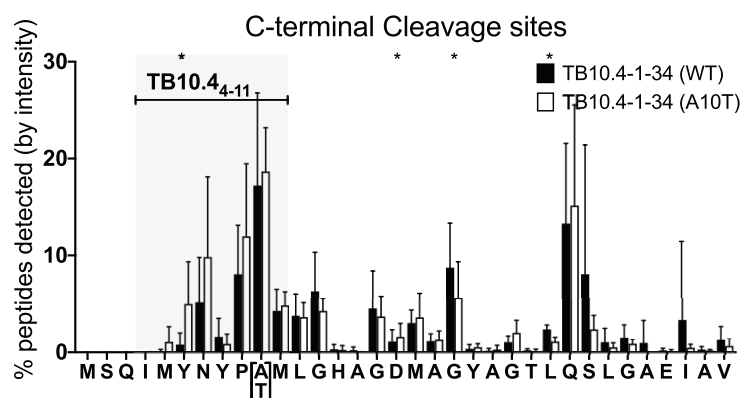

C

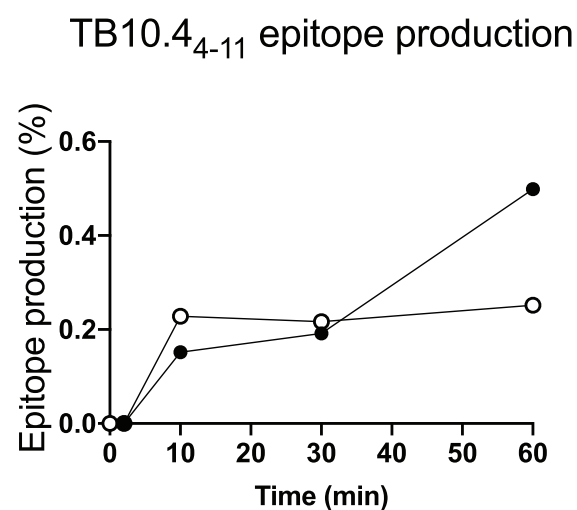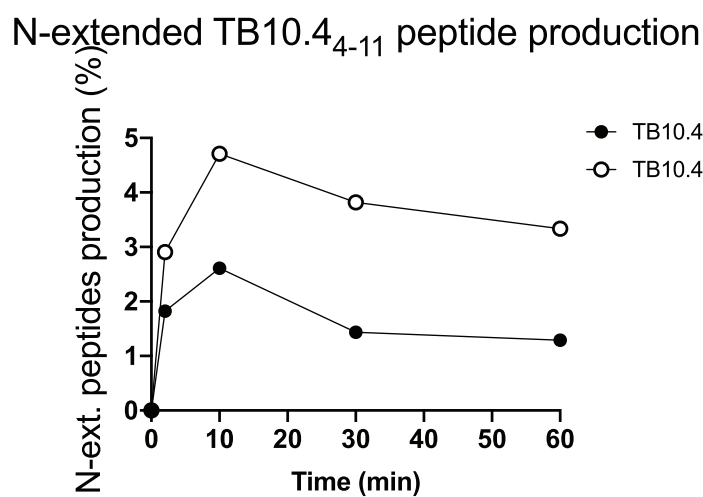

D

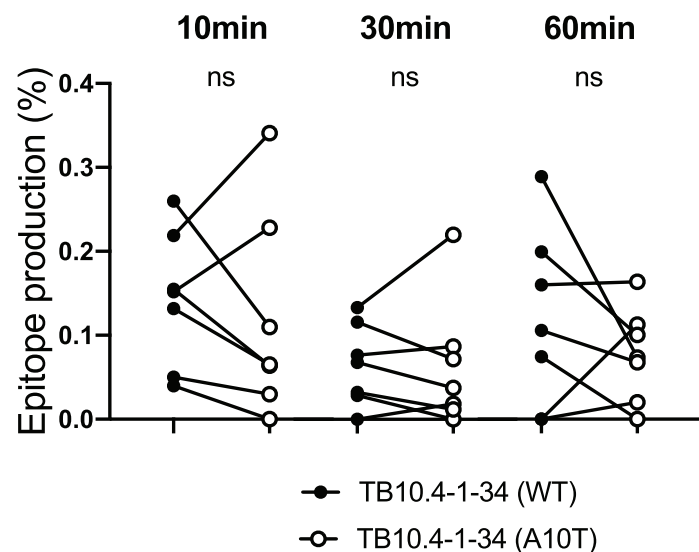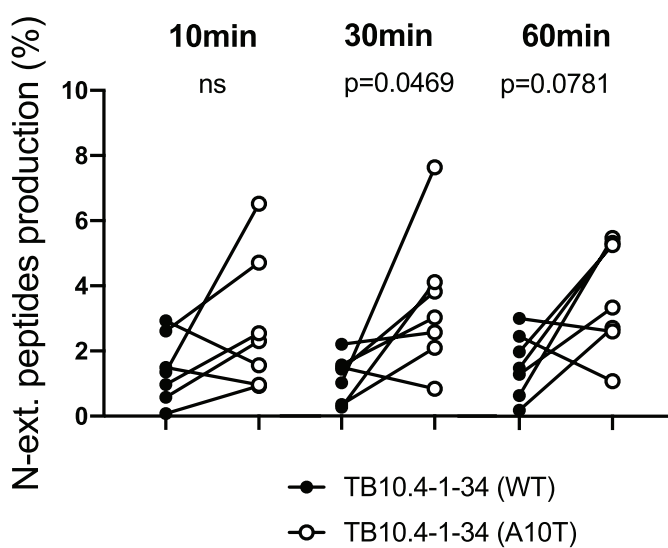

Supplement: S10 Fig — (A) Experiment scheme of the in vitro peptide degradation assay in bone-marrow derived dendritic cells lysosomal extracts. (B) N (left) and C (right) terminus cleavage sites determined at 60 minutes. The relative amount of peptides starting (left) or ending (right) at each residue was quantified during the degradation of WT (closed) and A10T (open) TB10.4-1-34-mer peptides. N = 6 experiments; stars p<0.05. (C) Production of TB10.44−11 epitope (left) and N-extended IM8 (right) from WT (closed) or A10T 34-mer (open) at 60 minutes. One representative experiment. (D) Production of TB10.44−11 epitope from WT (closed) and A10T (open) TB10.4-1-34-mer peptides was determined at 10, 30, 60 minutes in N = 6 independent experiments. P values calculated with Wilcoxon matched-pairs signed rank test. (PDF) [file ppat.1009000.s010.pdf]
