# Supplementary material for: A natural polymorphism of Mycobacterium tuberculosis in the esxH gene disrupts immunodomination by the TB10.4-specific CD8 T cell response
Source: PLoS Pathog. 2020 Oct 19;16(10):e1009000. doi: 10.1371/journal.ppat.1009000 (PMC7597557; doi:10.1371/journal.ppat.1009000)
Supplement: S9 Fig — Normalized and fitted data from Fig 6A.using the ECanything nonlinear regression model (Prism 8) to fit the curves. By setting F = 50, the IC50 was determined for each data set. The points are the experimental data, the solid lines are the fitted line, and the dashed lines are the 95% confidence intervals. This experiment was repeated four times with similar results. (PDF) [file ppat.1009000.s009.pdf]

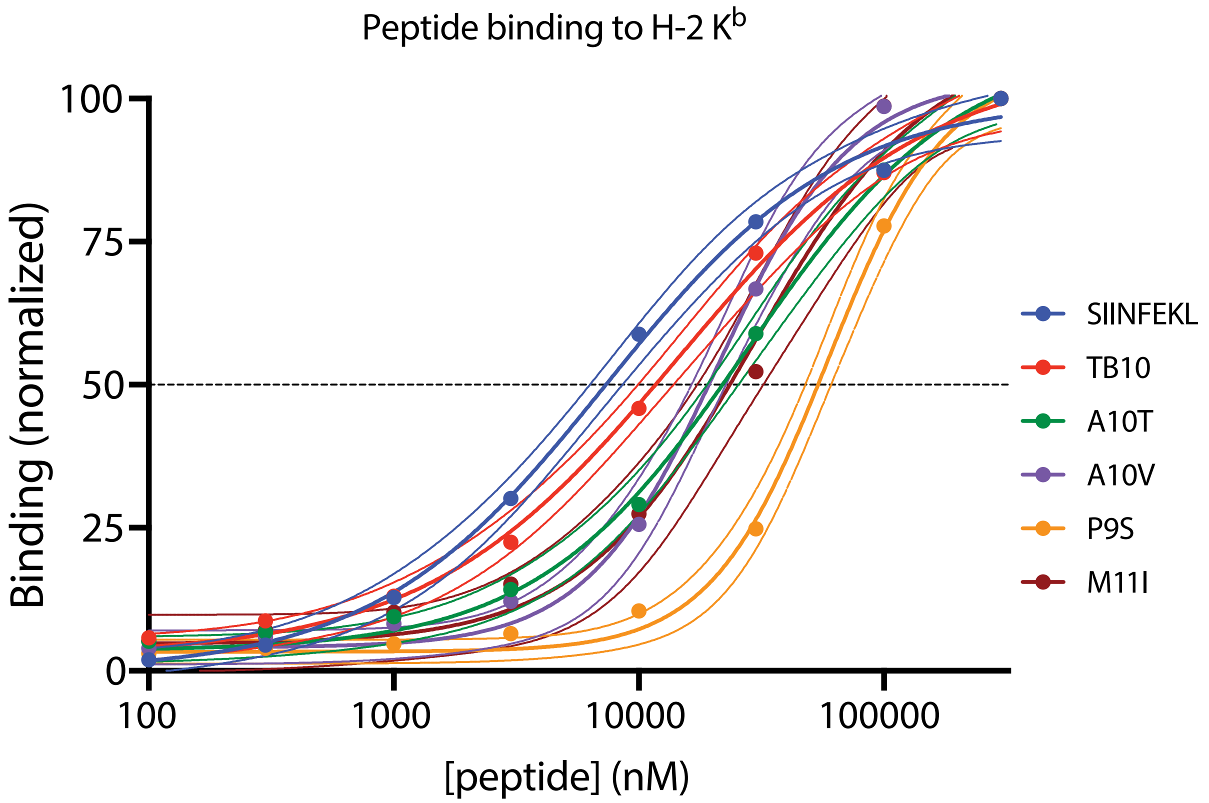

	SIINFEKL	TB10	A10T	A10V	P9S	M11I	IMANAPAM
[Agonist] vs. response – Find ECanything							Ambiguous
Best-fit values							
ECF	7318	14363	28078	21278	59349	29705	~ 115602
F	= 50.00	= 50.00	= 50.00	= 50.00	= 50.00	= 50.00	= 50.00
HillSlope	0.9188	0.8498	1.008	1.593	1.807	1.222	~ 8.816
Bottom	-0.1011	2.707	3.466	4.088	3.341	4.703	15.19
Top	99.97	106.3	109.7	103.6	105.6	110.3	100
logECF	3.864	4.157	4.448	4.328	4.773	4.473	~ 5.063
Span	100.1	103.6	106.2	99.56	102.3	105.6	84.83
Goodness of Fit							
Degrees of Freedo	7	7	7	7	7	7	7
R squared	0.9979	0.9974	0.9973	0.9956	0.9967	0.987	0.8943
Sum of Squares	32.71	36.56	35.27	67.72	39.22	185.8	775.6
Sy.x	2.162	2.285	2.245	3.11	2.367	5.152	10.53
Constraints							
F	*F = 50*	F = 50	F = 50	F = 50	F = 50	F = 50	F = 50
Number of points							
# of X values	11	11	11	11	11	11	11
# Y values analyzed	11	11	11	11	11	11	11
