# Supplementary material for: A natural polymorphism of Mycobacterium tuberculosis in the esxH gene disrupts immunodomination by the TB10.4-specific CD8 T cell response
Source: PLoS Pathog. 2020 Oct 19;16(10):e1009000. doi: 10.1371/journal.ppat.1009000 (PMC7597557; doi:10.1371/journal.ppat.1009000)
Supplement: S1 Table — The number of each polymorphism was indicated among different Mtb lineages with the number of independent occurrences of each polymorphism in parentheses. (PDF) [file ppat.1009000.s011.pdf]

Table S1: Identification of *esxH* nonsynonymous polymorphisms among clinical isolates. The number of each polymorphism was indicated among different Mtb lineages with the number of independent occurrences of each polymorphism in parentheses.

Polymorphisms	Lineage 1	Lineage 2	Lineage 3	Lineage 4
	Number Isolates (Number evolved)			
N7D	2 (1)			
N6S	2 (1)			
P9S	17 (1)			
A10P	1 (1)			
A10T	41 (1)			
A10V	6 (3)	1 (1)		4 (1)
M11L	1 (1)			
L12F	1 (1)			
G13D	1 (1)			
T24K		1 (1)		
Q57L	1 (1)			
A61V	1 (1)			
H70Y	1 (1)[a]			1 (1)
S74R	4 (1)[b]	1 (1)		
T75A		1 (1)		
T75S				1 (1)
H76L	1 (1)			
A89S				1 (1)
Total	75/594	4/1239	0/529	7/1001
Percent	12.6%	0.3%	0.0%	0.7%

[a]Also A10V mutant
[b]Also A10T mutants
