# Supplementary material for: A natural polymorphism of Mycobacterium tuberculosis in the esxH gene disrupts immunodomination by the TB10.4-specific CD8 T cell response
Source: PLoS Pathog. 2020 Oct 19;16(10):e1009000. doi: 10.1371/journal.ppat.1009000 (PMC7597557; doi:10.1371/journal.ppat.1009000)
Supplement: S2 Table — (PDF) [file ppat.1009000.s012.pdf]

Table S2: Peptide sequences used in this study.

Peptides	Sequences
TB10	IMYNYPAM
A10T	IMYNYPTM
A10V	IMYNYPVM
P9S	IMYNYSAM
M11I	IMYNYPAI
Nonbinder	IMANAPAM
Positive	SIINFEKL
TB10.4: 1 of 21	MSQIMYNYPAMLGHA
TB10.4: 2 of 21	MYNYPAMLGHAGDMA
TB10.4: 3 of 21	PAMLGHAGDMAGYAG
TB10.4: 4 of 21	GHAGDMAGYAGTLQS
TB10.4: 5 of 21	DMAGYAGTLQSLGAE
TB10.4: 6 of 21	YAGTLQSLGAEIAVE
TB10.4: 7 of 21	LQSLGAEIAVEQAAL
TB10.4: 8 of 21	GAEIAVEQAALQSAW
TB10.4: 9 of 21	AVEQAALQSAWQGDT
TB10.4: 10 of 21	AALQSAWQGDTGITY
TB10.4: 11 of 21	SAWQGDTGITYQAWQ
TB10.4: 12 of 21	GDTGITYQAWQAQWN
TB10.4: 13 of 21	ITYQAWQAQWNQAME
TB10.4: 14 of 21	AWQAQWNQAMEDLVR
TB10.4: 15 of 21	QWNQAMEDLVRAYHA
TB10.4: 16 of 21	AMEDLVRAYHAMSST
TB10.4: 17 of 21	LVRAYHAMSSTHEAN
TB10.4: 18 of 21	YHAMSSTHEANTMAM
TB10.4: 19 of 21	SSTHEANTMAMMARD
TB10.4: 20 of 21	EANTMAMMARDTAEA
TB10.4: 21 of 21	MAMMARDTAEAAKWGG
MTB32a 309-318	GAPINSATAM
ESAT6 1-15	MTEQQWNFAGIEAAA
TB10.4 20-28	GYAGTLQSL
TB10.4 74-88	STHEANTMAMMARDT
EspA 150-158	AYLVVKTLI
