# Supplementary material for: A natural polymorphism of Mycobacterium tuberculosis in the esxH gene disrupts immunodomination by the TB10.4-specific CD8 T cell response
Source: PLoS Pathog. 2020 Oct 19;16(10):e1009000. doi: 10.1371/journal.ppat.1009000 (PMC7597557; doi:10.1371/journal.ppat.1009000)
Supplement: S3 Table — Bold letters indicate polymorphic substitution. (PDF) [file ppat.1009000.s013.pdf]

Table S3: Predicted processing efficiency and binding affinity to H2-K^b of polymorphic TB10.4$_{4-11}$ epitopes. Bold letters indicate polymorphic substitution

Variants	15mer	epitope	Proteasome score	TAP score	MHC score	Processing score	Total score	MHC ic50
WT	IMYNYPAMLGHAGDM	IMYNYPAM	0.94	0.23	-0.87	1.17	0.30	7.4
P9S	IMYNY**S**AMLGHAGDM	IMYNY**S**AM	0.82	0.23	-0.75	1.05	0.30	5.6
A10T	IMYNYP**T**MLGHAGDM	IMYNYP**T**M	1.07	0.23	-0.81	1.30	0.49	6.5
A10V	IMYNYP**V**MLGHAGDM	IMYNYP**V**M	1.09	0.23	-0.98	1.32	0.35	9.5
M11I	IMYNYPA**I**LGHAGDM	IMYNYPA**I**	1.07	0.33	-0.88	1.40	0.52	7.6
Nonbinder	IM**A**N**A**PAMLGHAGDM	IM**A**N**A**PAM	0.94	0.15	-3.13	1.10	-2.04	1356.5
